# Enabling Peptide Ligation at Aromatic Junction Mimics via Native Chemical Ligation and Palladium-Mediated S-Arylation

**DOI:** 10.1021/acs.orglett.3c01652

**Published:** 2023-06-15

**Authors:** Xiaoxi Lin, Raj V. Nithun, Raju Samanta, Omer Harel, Muhammad Jbara

**Affiliations:** School of Chemistry, Raymond and Beverly Sackler Faculty of Exact Sciences, Tel Aviv University, Tel Aviv 69978, Israel

## Abstract

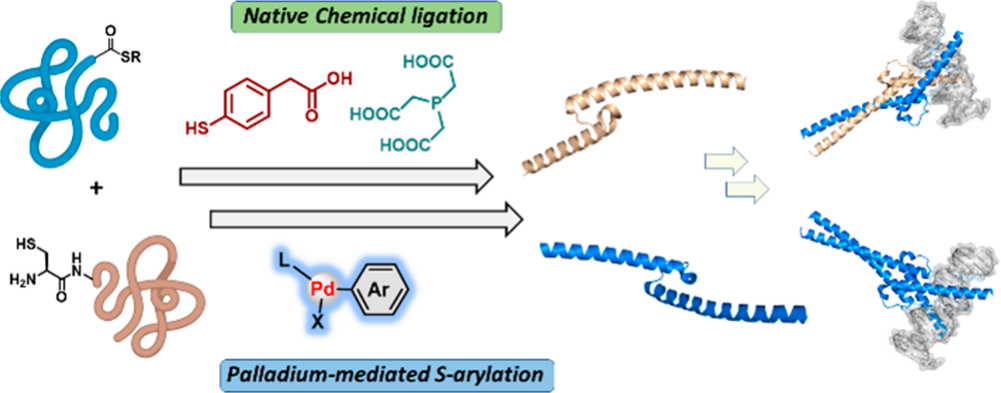

Synthetic strategies to assemble peptide fragments are in high demand to access homogeneous proteins for various applications. Here, we combined native chemical ligation (NCL) and Pd-mediated Cys arylation to enable practical peptide ligation at aromatic junctions. The utility of one-pot NCL and S-arylation at the Phe and Tyr junctions was demonstrated and employed for the rapid chemical synthesis of the DNA-binding domains of the transcription factors Myc and Max. Organometallic palladium reagents coupled with NCL enabled a practical strategy to assemble peptides at aromatic junctions.

Chemical protein synthesis provides powerful means to produce synthetic proteins for a variety of applications, ranging from mechanistic investigations to the development of novel bioactive compounds.^1,2^ This has been achieved by combining a set of synthetic strategies that predominantly capitalize on solid-phase peptide synthesis (SPPS) and chemoselective ligation technology.^3^ Such a combination, which is often combined with postsynthetic transformations, enables the production of synthetic proteins in a rigorous manner. SPPS allows for the incorporation of the desired residue during peptide elongation at the solid support. Then, synthetic peptides are assembled in solution through peptide ligation reactions to afford the target protein.^3^ Several ligation approaches have been developed in the past decade.^4,5^ In this regard, native chemical ligation (NCL)^6^ and other innovative extended methods have been widely employed to produce homogeneous proteins.^4^ In this reaction, peptide segments bearing an N-terminal Cys and a C-terminal thioester are reacted in solution through a *trans*-thioesterification step, followed by an intramolecular S- to N-acyl shift to provide a native amide bond. This approach enables the synthesis of medium-to-large proteins with post-translational modifications (PTMs) as well as noncanonical tags for various applications.^1−3^

Significant effort has been invested in the past years to expand the scope of NCL-based protein synthesis to enable peptide ligation beyond the Cys junction.^4^ Early reports introduced metal- and radical-based desulfurization strategies to convert Cys at the ligation site to Ala after NCL.^7,8^ The successful use of NCL–desulfurization chemistry at the Ala junction has triggered the development of several thiolated amino acids,^9^ which have been incorporated at the N-terminus of peptide fragments for subsequent NCL–desulfurization.^10^ In addition, selenocysteine-based NCL coupled with deselenization has also been investigated.^11,12^ Although significant developments have been made to expand the scope of NCL using noncanonical thiolated amino acids, access to these building blocks remains a challenge. Furthermore, protein synthesis via NCL at aromatic junctions has been rarely explored.^13,14^

Site-selective bioconjugation chemistry enables the well-defined covalent attachment of small molecules to biomolecules.^15,16^ The low abundance and high nucleophilicity of the thiol residue of Cys under physiological conditions make Cys the most suitable site for protein modifications.^17,18^ Among these reactions, Cys arylation has shown a promising capacity to functionalize biomolecules.^19^ In this regard, Cys arylation with palladium(II) oxidative addition complexes (Pd-OACs) is of particular interest due to the high reaction rate and the stability of the S-aryl linkages formed.^20^ We envisioned that such organometallic complexes could be employed for chemical protein synthesis by enabling the rapid substitution of the Cys residue at the ligation site to an aromatic residue mimic. Interestingly, previous Cys alkylation methods used to install aliphatic side chains have shown that such alteration is a reasonable mimic of native residues.^21,22^ Herein, we combined NCL and palladium-mediated C–S arylation to enable practical peptide ligation at aromatic junctions. Palladium-mediated cross-coupling of ligated peptide fragments furnished aromatic junction mimics within minutes in an aqueous buffer. We employed our approach in one-pot NCL and S-arylation at Phe and Tyr, enabling the efficient preparation of functional DNA-binding domains of the transcription factors (TFs) Myc and Max from two segments. Combining chemical protein synthesis and palladium-mediated C–S arylation enabled practical peptide ligation at aromatic junctions.

We initially designed our organometallic palladium reagent to transfer aromatic residue mimics under water-compatible conditions. We combined a bis[(trimethylsilyl)methyl](1,5-cyclooctadiene)palladium(II) [(COD)Pd(CH_2_TMS)_2_] precursor with target haloarene derivatives following the reported protocol of Buchwald and co-workers.^20^ We prepared our complexes in the presence of a water-compatible sulfonated biarylphosphine ligand (sSPhos)^23^ to provide complexes **Pd-1** and **Pd-2** in 97% isolated yield (Figure 1A). We then tested the reactivity of these complexes with a nine-mer model peptide (YRAGCYRAG, **P1**), prepared using a standard Fmoc-SPPS protocol. We initially treated **P1** with **Pd-1** and **Pd-2** (5 equiv) separately in 20 mM Tris buffer (pH 7.2) at room temperature. This led to quantitative conversion to the desired S-arylated product within 15 min, as determined by high-performance liquid chromatography–mass spectrometry (HPLC–MS) analysis (Figure 1B–D). These findings demonstrate that both reagents could transfer the desired aromatic residues (e.g., Tyr and Phe mimics) to Cys-containing polypeptides in a rapid and effective manner (Figure 1C). Despite several attempts to expand this approach to transfer the Trp residue mimic, we could not isolate the desired indole-based Pd-OAC **Pd-3** using different haloindole analogues and ligands, e.g., SPhos or RuPhos.^24^

**Figure 1:**
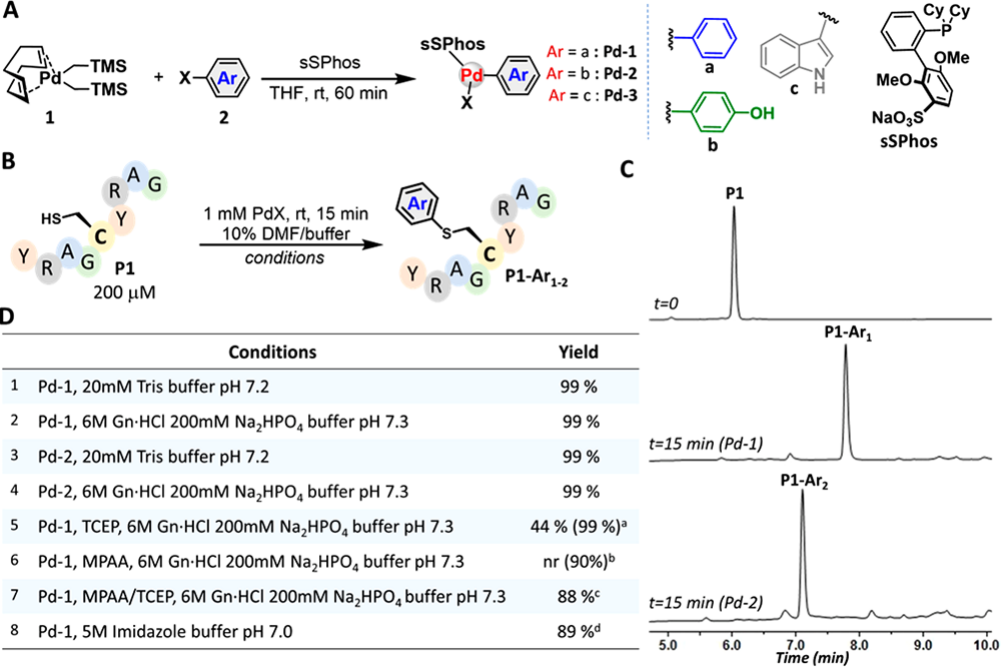
Pd-1 and Pd-2 enable effective transfer of aromatic residue mimics under NCL conditions. (A) Synthesis of the Pd-OACs. (B) S-arylation of the model peptide P1. (C) Representative analytical RP-HPLC analysis of the arylation of P1 with Pd-1 and Pd-2, respectively. (D) Table summarizing the S-arylation conversion yields. *Yields are based on the integration of the crude conjugation reaction from RP-HPLC analysis. ^a^Reaction was performed using 2 mM Pd-1, ^b^Oxidized product, with MPAA. ^c^Reaction was performed using 4 mM Pd-1, and ^d^Reaction was performed using 2 mM Pd-1 at 37 °C for 40 min.

We then set out to explore the reactivity of **Pd-1** and **Pd-2** under NCL conditions, in particular, in chaotropic buffers, under high concentrations of guanidine hydrochloride (Gun.HCl), and in the presence of ligation additives (i.e., thiols and phosphines). For this, we first exposed **P1** to 5 equiv of **Pd-1** in 6 M Gun.HCl and 200 mM Na_2_HPO_4_ buffer (pH 7.3). HPLC–MS analysis confirmed the formation of the desired product with ∼99% conversion after 15 min (Figure 1D). The same outcome was observed when transferring a Tyr mimic using **Pd-2** under the same conditions. These findings demonstrate the compatibility of Pd-OAC chemistry under denaturation conditions, which is known to chelate Pd(II) in aqueous buffer and potentially affect its reactivity.^25^ Exposing **P1** to **Pd-1** in the presence of the reducing agent tris(2-carboxyethyl)phosphine (TCEP) furnished the desired S-aryl product, although in 40% conversion. We attributed the diminished activity to potential phosphene-ligand exchange; e.g., TCEP/sSPhos, which can affect **Pd-1** composition and reactivity. This could be overcome by increasing the **Pd-1** loading to 10 equiv to furnish the desired product in 99% conversion after 15 min. Next, we tested the same reaction in the presence of the aryl thiol additive 4-mercaptophenylacetic acid (MPAA), which is frequently used in NCL to catalyze peptide ligation. However, under these conditions, no product was observed, and we mainly observed peptide–MPAA oxidized byproduct. Remarkably, exposing **P1** to **Pd-1** in the presence of both MPAA and TCEP additives furnished the desired product with 88% conversion. Finally, the same reactivity was observed when using the non-thiol-type NCL additive imidazole, which can function as an alternative to MPAA.^26^

Encouraged by these results, we then sought to explore the potential of our strategy in chemical protein synthesis when coupled with in situ NCL. We first employed our approach to synthesize a model polypeptide from two segments by ligating the model peptide (CHSLRDSVPSLQ, **3**) with a model peptide thioester (VYKSPLYKSR-SR, **4**). Both peptide segments were prepared using a standard SPPS protocol (see SI). Segment **3** was modified with the N-terminal Cys residue to enable NCL at this site. The peptide thioester **4** was prepared using the N-acylurea method, starting with Dawson’s linker (3,4-diaminobenzoic acid, Dbz).^27^ Next, peptides **3** and **4** were ligated under NCL conditions, which furnished the desired product after 30 min (Figure 2). Subsequently, we reacted crude reaction intermediate **5** with **Pd-1** to convert Cys at the ligation site to a Phe mimic. We obtained the desired S-arylated product after 30 min as confirmed via HPLC–MS analysis. Finally, the reaction mixture was quenched by the addition of 3-mercaptopropionic acid (3-MPA) and purified via reverse phase (RP)-HPLC to provide the desired product in 57% isolated yield for both synthetic steps. These findings demonstrate the compatibility of Pd-OACs with NCL conditions, highlighting the potential of combining both approaches for chemical protein synthesis.

**Figure 2:**
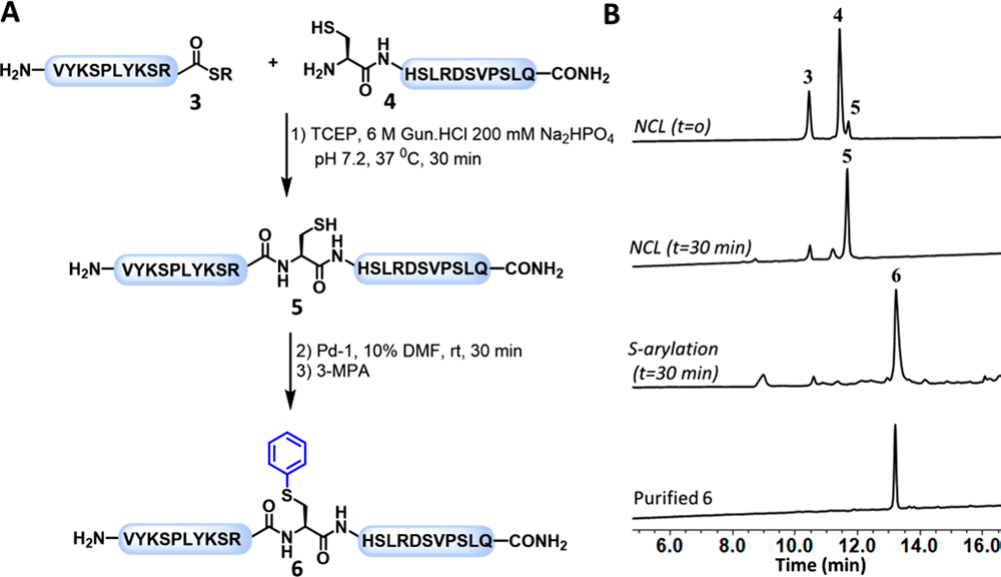
One-pot NCL and palladium-mediated C–S arylation. (A) Schematic representation of the ligation and one-pot S-arylation. (B) Analytical RP-HPLC analysis of the ligation and S-arylation reactions.

NCL and palladium-mediated C–S arylation enabled rapid production of the DNA-binding domains of Myc and Max. TFs are essential proteins for controlling gene expression by interacting with specific DNA sequences to initiate or suppress gene transcription. For example, the master regulator Myc controls the expression of ∼10% of the human genome, and its dysregulation is implicated in up to 70% of human tumors.^28^ To be active, Myc forms a heterodimeric complex with its cognate partner Max and binds to promoter regions that contain the enhancer box DNA sequence (E-box). By contrast, Max can self-dimerize (Max/Max) and bind a similar E-box sequence as Myc/Max but lacks the transactivation domain found in Myc, thus suppressing Myc-mediated gene transcription. Recent reports have shown that synthetic TFs hold promising potential to inhibit the oncogenic activity of Myc by competing for the same DNA sequence.^29,30^ Previous reports showed that the DNA-binding domains of Myc and Max could be prepared from three peptide fragments by applying sequential NCL and desulfurization or via flow-based protein synthesis.^29,31^ Here, we decided to employ our strategy for the chemical synthesis of Myc and Max DNA-binding domains from two fragments using the NCL and S-arylation approach. We first turned our attention to applying this strategy to facilitate the chemical synthesis of Max. We identified a potential ligation site at Ser–Phe located in the basic helix domain (Figure 3A). Therefore, we divided Max into two fragments (i.e., Max(22–42)-Nbz (**7**) and Cys-Max(44–102) (**8**) and synthesized both fragments via Fmoc-based SPPS. The N-terminal Phe43 residue in fragment **8** was mutated to Cys to enable NCL at this site. On the other hand, Max(22–42)-Nbz **7** was synthesized using a Dbz linker followed by on-resin cyclization to furnish the activated N-acyl urea moiety. We obtained segments **7** and **8** in 59 and 16% isolated yields, respectively, after RP-HPLC purification. Having both fragments in hand, we proceeded with the ligation, which was performed under standard NCL conditions using MPAA and TCEP additives. The ligation reaction was fast and completed within 1 h at 37 °C to furnish the desired ligated product, as confirmed via HPLC–MS analysis. Subsequently, we desalted the reaction mixture and subjected the crude ligated product to **Pd-1** to convert Cys at the ligation site to a Phe mimic to furnish the S-arylated product within 20 min. We obtained the final desired product Max in 25% overall isolated yield for the two steps (Figure 3B), which were operated in a one-pot manner.

**Figure 3:**
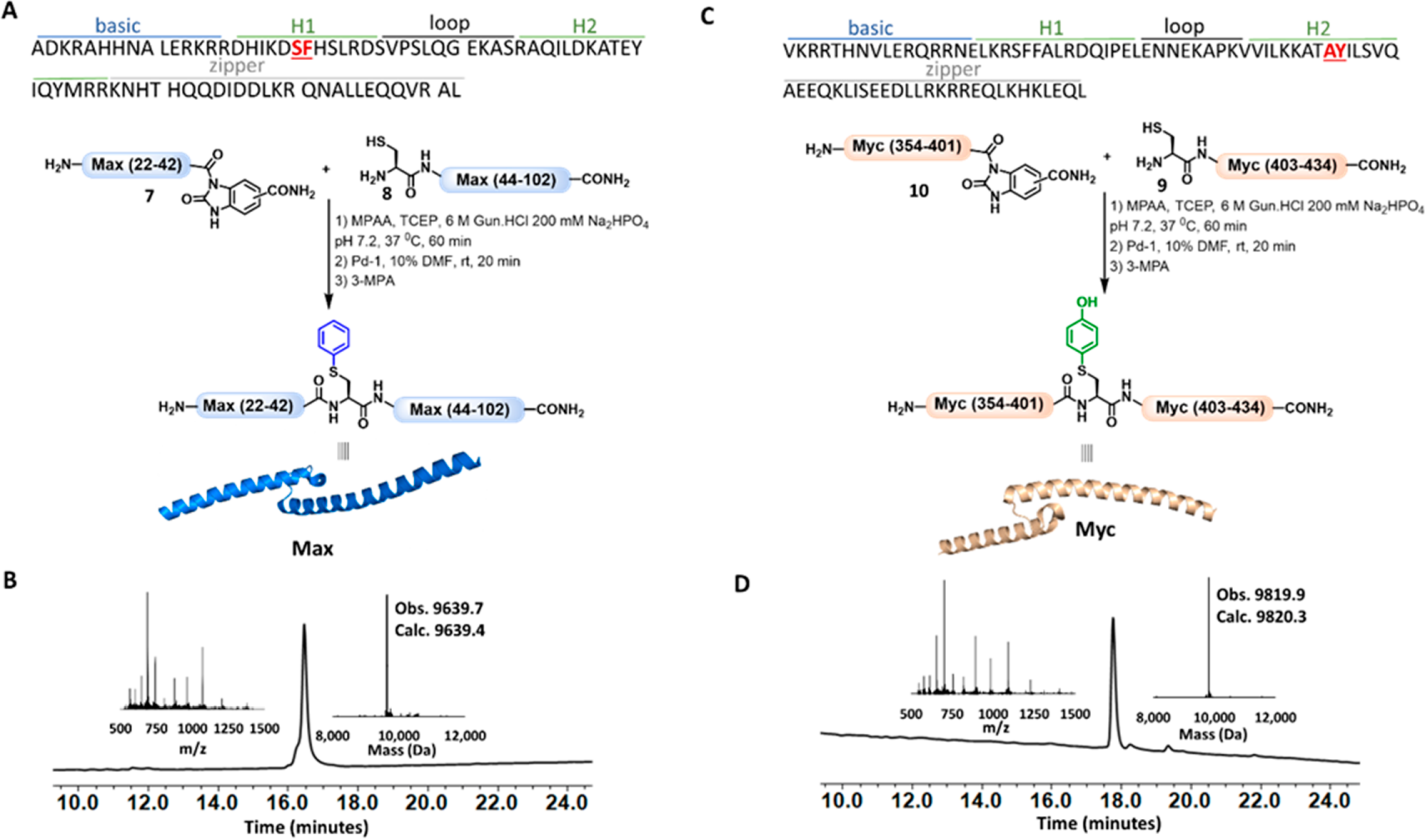
Chemical synthesis of Myc and Max via NCL and palladium-mediated S-arylation. (A) Schematic representation of the synthesis of Max. (B) HPLC–MS analysis of Max. (C) Schematic representation of the synthesis of Myc. (D) HPLC–MS analysis of Myc.

For Myc synthesis, we identified a potential ligation junction at the Ala–Tyr site located in the Lue zipper helix (Figure 3C). Like the synthesis of Max, we divided Myc into two fragments; i.e., Cys-Myc(403–434) (**9**) and Myc(354–401)-Nbz (**10**). However, we failed to obtain both fragments, despite several attempts using SPPS under different coupling conditions. After multiple analytical HPLC–MS analyses, we identified that the tetra-amino acid sequence (RXRR, X = Q in fragment **9** and X = K in fragment **10**) in both segments led to the reduction in the synthesis yield and quality. After screening different coupling reagents (e.g., HATU, HBTU, HCTU, and DIC), we found that shifting to HOBt/DIC led to a major improvement in both syntheses. We were able to isolate both fragments **9** and **10** in 32 and 10% yield, respectively. We then ligated both synthetic fragments under standard NCL conditions. Complete ligation of both starting materials was achieved within 1 h at 37 °C. Subsequently, we treated the crude reaction mixture with **Pd-2** to obtain the final product in a 23% overall isolated yield for the two steps (Figure 3D).

We next probed the DNA-binding activity of the synthesized proteins via an NCL and S-arylation approach (Figure 4A,B). We first incubated each one of the proteins separately with a 22 bp double-stranded DNA probe containing the canonical E-box sequence and examined the DNA-binding activity by an electrophoretic mobility-shift assay (EMSA). We found that synthetic Max efficiently associated with the E-box DNA probe as indicated by a significant upward shift in a dose–response manner (Figure 4B). As expected, we did not detect any DNA-binding activity with Myc to the E-box probe because Myc protein itself cannot homodimerize but binds efficiently when dimerized with Max. Finally, we combined equimolar concentrations of the synthetic Myc and Max analogues to form the heterodimeric Myc/Max complex. We found that the heterodimeric complex binds the E-box DNA probe. Next, we measured the dissociation constant of synthetic Max to the E-box DNA probe by biolayer interferometry (BLI). We determined a *K*_D_ of 14.8 ± 0.2 nM, which is in good agreement with previous reports for the recombinant Max and E-box complex.^32^ Importantly, these experiments together demonstrate that synthetic proteins prepared via NCL coupled with palladium-mediated S-arylation are functional and effectively bind the target DNA sequence.

**Figure 4:**
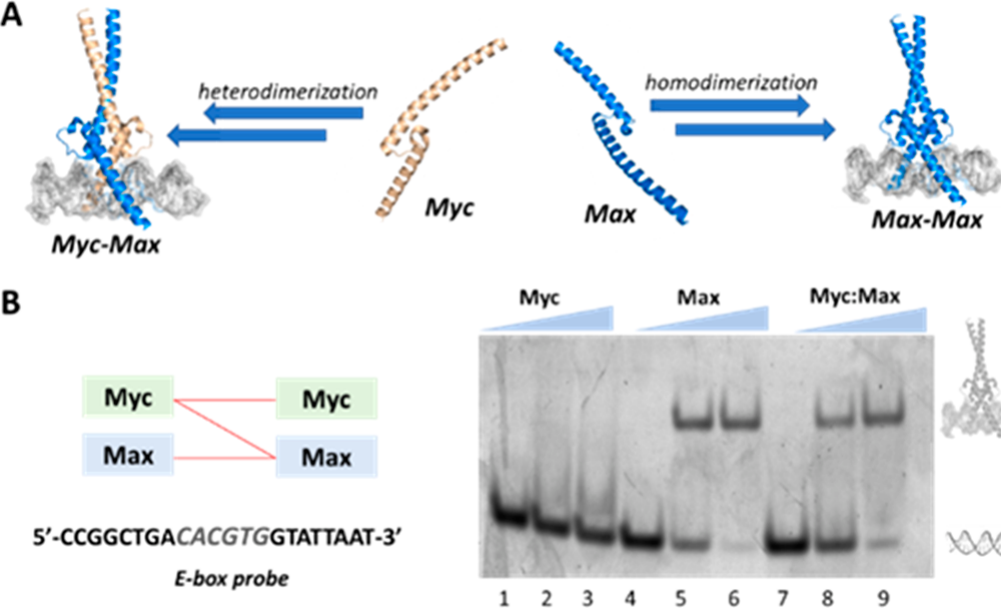
Synthetic Myc and Max are functional and capable of binding to canonical E-box DNA. (A) Schematic representation of Myc and Max dimerization and DNA-binding. (B) EMSA experiment of synthetic proteins. Conditions: 1 μM DNA probe and 0, 4, 8 μM protein.

In summary, we accomplished rapid NCL and palladium-mediated S-arylation at the Phe and Tyr junctions to furnish the desired proteins in good yield. We employed this approach for the total chemical synthesis of the DNA-binding domains of Myc and Max TFs, which exhibited potent DNA-binding activity to target the recognition sequence. Importantly, we extended the potential of Pd-OACs to chemical protein synthesis and showed its compatibility with NCL, highlighting the substantial potential of this approach for protein production. We envision this approach to enable chemoselective and rapid incorporation of noncanonical aromatic residues to generate well-defined modified proteins. This would enable the production of new artificial proteins with novel physicochemical properties and tuned activity for various potential applications.^33,34^ In addition to transition-metal-mediated protein bioconjugation,^35^ this work expands the scope of organometallic reagents for total chemical protein applications. Importantly, this strategy should enable rapid and effective peptide ligation at aromatic junctions when the use of thiol-derivatized amino acids or a desulfurization approach is not possible.

## Data Availability

The data underlying this study are available in the published article and its Supporting Information.

## Supporting Information Available

Experimental; synthesis of palladium(II) oxidative addition complexes (PdOACs); protein and DNA sequences; chemical synthesis of peptide fragments; S-arylation of peptide P1; native chemical ligation and one-pot S-arylation with PdOACs; DNA-binding analysis and electrophoretic mobility-shift assay (EMSA); biolayer interferometry binding assay (BLI); NMR data for PD-1 and PD-2 (PDF)
