# Supplementary material for: Enabling Peptide Ligation at Aromatic Junction Mimics via Native Chemical Ligation and Palladium-Mediated S-Arylation
Source: Org Lett. 2023 Jun 15;25(25):4715–9. doi: 10.1021/acs.orglett.3c01652 (PMC10324392; doi:10.1021/acs.orglett.3c01652)
Supplement: Supplementary file 1 — ol3c01652_si_001.pdf [file ol3c01652_si_001.pdf]

Supporting Information

Enabling Peptide Ligation at Aromatic Junction Mimics via Native Chemical Ligation and Palladium-Mediated S-Arylation

Xiaoxi Lin, Raj V. Nithun, Raju Samanta, Omer Harel, and Muhammad Jbara*

School of Chemistry, Raymond and Beverly Sackler Faculty of Exact Sciences, Tel Aviv University, Tel Aviv, 69978 Israel

*Correspondence to: jbaram@tauex.tau.ac.il

Table of Contents

1. EXPERIMENTAL **3**

1.1 Materials 3
1.2 Analytical reversed-phase high-pressure liquid-chromatography (RP-HPLC) 4
1.3 Preparative RP-HPLC purification 4

2. SYNTHESIS OF PALLADIUM(II) OXIDATIVE ADDITION COMPLEXES (PDOACS) **4**

3. PROTEIN AND DNA SEQUENCES **6**

4. CHEMICAL SYNTHESIS OF PEPTIDE FRAGMENTS **7**

4.1 Synthesis of model peptide (P1) 7
4.2 Synthesis of model peptide (3) 8
4.3 Synthesis of model peptide (4) 9
4.4 Max(22-42)-Nbz fragment (7) 11
4.5 Cys-Max(44-102) fragment (8) 12
4.6 Cys-Myc(403-434) fragment (9) 13
4.7 Myc(354-401)-Nbz fragment (10) 14

5. S-ARYLATION OF PEPTIDE P1 .... **16**

5.1 Evaluation of Reaction Conditions for Peptide Arylation 16

6. NATIVE CHEMICAL LIGATION AND ONE-POT S-ARYLATION WITH PDOACS **23**

6.1 One-pot NCL and S-arylation 23
6.2 Total chemical synthesis of Max via NCL and one-pot S-arylation 24
6.3 Total chemical synthesis of Myc via NCL and one-pot S-arylation 26
6.4 S-arylation of Max followed by byproduct removal via molecular weight filtration 28

7. DNA-BINDING ANALYSIS AND ELECTROPHORETIC MOBILITY-SHIFT ASSAY (EMSA) **30**

8. OCTET BIOLAYER INTERFEROMETRY BINDING ASSAY (BLI) **31**

9. NMR DATA FOR PD-1 AND PD-2 .... **32**

10. REFERENCES **38**

1. Experimental

1.1 Materials

Fmoc-L-Phe-OH, Fmoc-L-Asn(Trt)-OH, Fmoc-L-Gln(Trt)-OH, Fmoc-L-Arg(Pbf)-OH, Fmoc-L-Tyr(*t*Bu)-OH, Fmoc-L-Glu(O*t*Bu)-OH, Fmoc-L-Ala-OH, Fmoc-L-Leu-OH, Fmoc-L-His(Trt)-OH, Fmoc-L-Asp(O*t*Bu)-OH, Fmoc-L-Pro-OH, Fmoc-L-Cys(Trt)-OH, Fmoc-L-Lys(Boc)-OH, Fmoc-L-Ile-OH, Fmoc-L-Ser(*t*Bu)-OH, Fmoc-Gly-OH, Fmoc-L-Nle-OH, Boc-L-Val-OH, Boc-L-Ala-OH, Boc-L-Cys(Trt)-OH, 1,3-Diisopropylcarbodiimide (DIC), and SPhos ligand were purchased from Sigma-Aldrich. Fmoc-L-His(Boc)-OH was purchased from CEM. 1-[Bis(dimethylamino)methylene]-1H-1,2,3-triazolo[4,5-b]pyridinium 3-oxide hexafluorophosphate (HATU), (2-(1H-benzotriazol-1-yl)-1,1,3,3-tetramethyluronium hexafluorophosphate (HBTU), Oxyma Pure, HOBt HYDRATE were purchased from Luxembourg Bio Technologies Ltd. H-Rink Amide ChemMatrix resin was obtained from Biotage. Rink Amide ProTide (LL) resin was obtained from CEM. Oligonucleotides were purchased from Integrated DNA Technologies (IDT). Diethyl ether (Et_2O, 99.8% stabilized, ACS grade) was obtained from MACRON. Dichloromethane (CH_2Cl_2, ≥99.5% stabilized with 50 ppm Amylene) was obtained from CHEM-LAB. *N*,*N*-dimethylformamide (DMF, Peptide Synthesis-grade) and acetonitrile (ACN, LC/MS Grade) were purchased from J.T.Baker. Trifluoroacetic acid (TFA, ≥99% ReagentPlus®), diisopropylethylamine (DIEA, ≥99% ReagentPlus®), piperidine (≥99% ReagentPlus®), triisopropylsilane (TIS, 98%), formic acid (FA, 98-100% for LC/MS), and dimethyl sulfoxide (DMSO, ≥99.5% ReagentPlus®) were purchased from Bio-Lab ltd. 10% TBE Gel (1.0 mm x 10 well), TBE Running buffer (5X), SYBR™ Safe DNA Gel Stain, pre-stained Invitrogen Orange DNA Loading Dye (6X) were purchased from Thermo Fisher Scientific. Water for all reactions carried out on proteins and for reverse-phase purification was obtained via filtration of deionized water through a MilliporeSigmaTM Milli-Q™ Ultrapure Water System. Deuterated Methanol-d_4 99.8% was purchased from ZEOtope. All chemicals obtained from supplier were used as received without further purification.

1.2 Analytical reversed-phase high-pressure liquid-chromatography (RP-HPLC)

Analytical RP-HPLC chromatograms were acquired using Thermo Scientific Vanquish HPLC. Mobile phases used are solvent A (0.05% TFA in water) and solvent B (0.05% TFA in acetonitrile).

Method A: Jupiter® 5 µm-C4 300 Å LC column (150 x 4.6 mm); LC conditions: 5% B from 0–1.0 min, then linear gradient from 5% to 60% B from 1.0–11.0 min, 1.0 mL/min flow rate.

Method B: Jupiter® 5 µm-C4 300 Å LC column (150 x 4.6 mm); LC conditions: 5% B from 0–1.0 min, then linear gradient from 5% to 60% B from 1.0–28.0 min, 1.0 mL/min flow rate.

Method C: XBridge® BEH 3.5 µm-C4 300 Å Column (150 x 4.6 mm); LC conditions: 5% B from 0–1.0 min, then linear gradient from 5% to 60% B from 1.0–28.0 min, 1.0 mL/min flow rate.

Mass spectra were acquired using Thermo Scientific ISQ EM Mass spectrometer.

1.3 Preparative RP-HPLC purification

Preparative RP-HPLC was performed using Thermo Scientific DIONEX UltiMate 3000 Variable Wavelength Detector, equipped with a Jupiter® 5 µm C18 300 Å LC column (250 x 10 mm) or Jupiter® 5 µm C4 300 Å LC column (250 x 10 mm). Mobile phases used for LC analysis were solvent A (0.05% TFA in water), solvent B (0.05% TFA in acetonitrile). The following LC methods were used:

Method A: Jupiter® 5 µm C4 300 Å LC column (250 x 10 mm), LC conditions: 5% B from 0–5 min, then linear gradient from 5% to 40% B from 5-40 min, 4 mL/min flow rate at 30 ^{o}C.

Method B: Jupiter® 5 µm C4 300 Å LC column (250 x 10 mm), LC conditions: 5% B from 0–5.0 min, then linear gradient from 5% to 60% B from 5-65 min, 4 mL/min flow rate at 30 ^{o}C.

Method C: Jupiter® 5 µm C18 300 Å LC column (250 x 10 mm), LC conditions: 5% B from 0–5.0 min, then linear gradient from 5% to 60% B from 5-65 min, 4 mL/min flow rate at 30 ^{o}C.

2. Synthesis of palladium(II) oxidative addition complexes (PdOACs)

Synthesis of sSPhos. This compound was prepared according to literature procedure.[1, 2] The ^{1}H and ^{31}P NMR spectra of the obtained material were identical to those reported in the literature.

Synthesis of [(COD)Pd(CH_2TMS)$_2$]. This compound was prepared according to literature procedure.[3] The ^{1}H NMR spectrum of the obtained material was identical to that reported in the literature.

Synthesis of PdOACs (Pd-1 and Pd-2): These two compounds were prepared according to literature procedure.[1, 2]

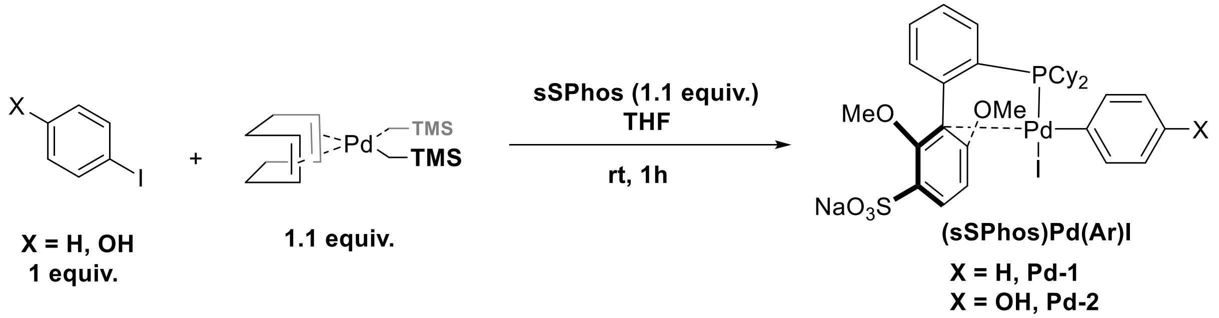

In a 20-mL scintillation vial which was opened to the air and equipped with a magnetic stir bar, ligand sSPhos (0.055 mmol, 1.1 equiv.) and Ar-I (0.05 mmol, 1 equiv.) were dissolved in dry THF (1 mL). Solid (COD)Pd(CH_2SiMe_3)$_2$ (0.055 mmol, 1.1 equiv.) was added rapidly in one portion. The reaction mixture was stirred for 1h at room temperature. After that, pentane (3 mL) was added, and the precipitated solid was collected by centrifuge (4000 rpm) for 4 min and washed with pentane (3 mL x 5). The brown solid was dried under vacuum. Pd-1 (40.1 mg, yield 97%) and Pd-2 (40.9 mg, yield 97.5%).

Note: We stored our complexes at room temperature under nitrogen atmosphere which gave no diminished reactivity even after 3 months.

Pd-1:

^{1}H NMR (400 MHz, MeOD) Complicated spectrum; please see the attached ^{1}H NMR spectrum. ^{13}C{1H} NMR (101 MHz, DMSO) δ 158.03 (s), 155.32 (s), 136.63 (s), 135.01 (s), 134.32 (s), 133.17 (s), 132.04 (s), 131.67 (s), 130.12 (s), 129.20 (s), 128.82 (s), 127.92 (s), 126.76 (s), 126.65 – 126.59 (m), 126.58 (s), 67.20 (s), 60.83 (s), 60.22 (s), 55.69 (s), 36.48 (s), 36.39 (s), 35.73 (s), 35.13 (s), 34.97 (s), 27.24 (s), 27.14 (s), 27.05 (s), 26.22 (s), 26.11 (s), 26.01 (s), 25.83 (s), 25.79 (s), 25.57 (s), 25.31 (s). (observed complexity is due to C–P coupling). ^{31}P NMR (162 MHz, MeOD) δ 54.2 (s). HRMS (ESI) m/z: [M – I]$^+$ calcd. for $C_{32}H_{39}NaO_5PPd^{[105]}S$: 694.1204, found 694.1217.

Pd-2:

^{1}H NMR (400 MHz, MeOD) Complicated spectrum; please see the attached ^{1}H NMR spectrum. ^{13}C{1H} NMR (101 MHz, DMSO) δ 158.03 (s), 155.34 (s), 140.62 (s), 138.04 (s), 137.24 (s), 136.25 (s), 135.51 – 135.24 (s), 135.06 (s), 134.54 (s), 133.29 (s), 133.22 (s), 133.10 (s), 130.08 (s), 129.26 (s), 126.75 (s), 126.66 (s), 124.78 (s), 115.39 (s), 105.36 (s), 105.07 (s), 60.85 (s), 55.69 (s), 37.09 (s), 36.66 (s), 36.43 (s), 36.00 (s), 27.02 (s), 26.42 (s), 26.36 (s), 26.13 (s), 25.89 (s), 25.89 (s), 25.58 (s), 25.46 (s). (observed

complexity is due to C–P coupling). ^{31}P NMR (162 MHz, MeOD) δ 51.6 (s). HRMS (APCI) m/z: $[M – Na]^-$ calcd. for $C_{32}H_{39}O_6PIPd^{[104]}S$: 813.0290, found 813.0273.

3. Protein and DNA sequences

Model peptide (P1): YRAGCYRAG

Model peptide (3): CHSLRDSVPSLQ

Model peptide (4): VYKSPLYKSR

Max(22-102):

ADKRAHHNALERKRRDHIKDS**F**HSLRDSVPSLQGEKASRAQILDKATEYIQYMRRKNHTHQQDI DDLKRQNALLEQQVRAL

The Phe residue at position 43 was mutated to Cys to enable native chemical ligation.

The Met residue at position 74 was mutated with the homologous norleucine (Nle) residue to avoid Met oxidation.

Myc(354-434):

VKRRTHNVLERQRRNELKRSFFALRDQIPELENNEKAPKVVILKKATA**Y**ILSVQAEEQKLISEEDL LRKRREQLKHKLEQL

The Tyr residue at position 402 was mutated to Cys to enable native chemical ligation.

E-box DNA probe:

5’-CCGGCTGA*CACGTG*GTATTAAT-3’

4. Chemical synthesis of peptide fragments

4.1 Synthesis of model peptide (P1)

The synthesis was carried out according to the following scheme:

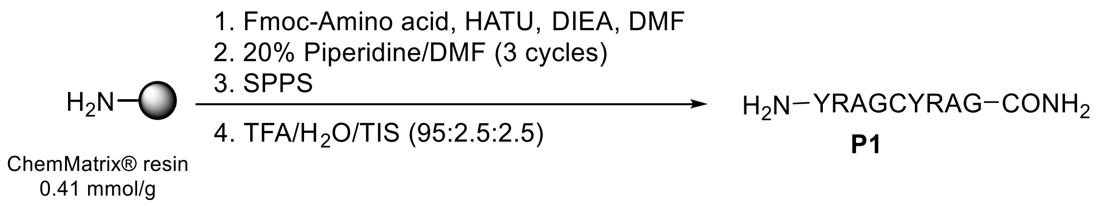

The synthesis of the model peptide P1 was carried out using Fmoc-SPPS on Rink amide ChemMatrix® resin (250 mg, loading 0.41 mmol/g, 0.1 mmol scale). The resin was pre-swollen with DMF for 15 min and all amino acids were coupled manually with Fmoc-amino acid (10 equiv.), using HATU (10 equiv.) and 0.5 mL DIEA in 2.5 mL DMF for 20 min. After each coupling step, the resin was washed 3 times with DMF and submitted to Fmoc-deprotection using 4 mL 20% piperidine/DMF containing 0.05% FA for 1 min, then 4 min, and another 1 min. After coupling all the amino acids, the peptide bound resin was washed with DMF (10 mL x 3) and DCM (10 mL x 3) and dried under vacuum. Subsequently a mixture of TFA/H_2O/TIS (95:2.5:2.5, 8 mL for 0.1 mmol scale) was added to the resin which was shaken for 1.5 h at room temperature. The resin was removed by filtration and washed with TFA (2 mL x 1). To precipitate the peptide, the combined filtrate was added dropwise to cold diethyl ether (35 mL for 0.025 mmol resin) followed by centrifugation at 4000 rpm for 4 min. Then, the diethyl ether was decanted, followed by dissolution of the peptide in 25% acetonitrile/water and lyophilized to get white powder. The crude dry peptide powder was purified by RP-HPLC (Method A that described in Section 1.3) affording the product 64.8 mg as white powder (yield 63%).

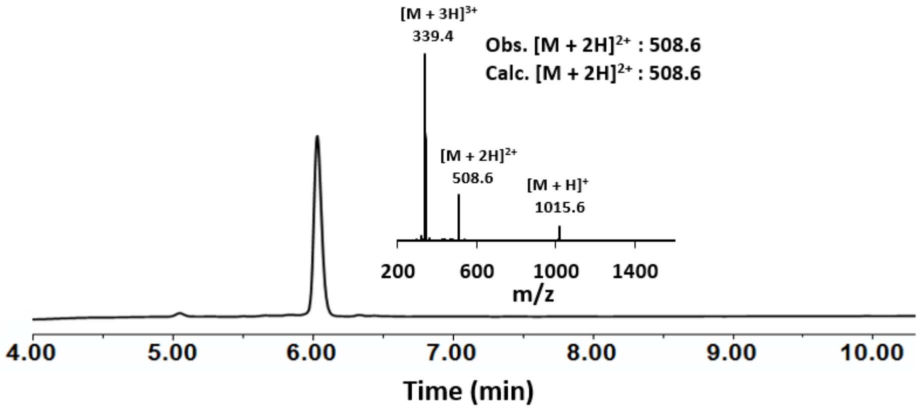

Figure S1. HPLC and MS analysis of model peptide (P1). HPLC chromatogram of the UV absorbance at 214 nm. HPLC analysis was carried out with Method A (see section 1.2). Mass-to-charge (m/z) spectrum.

4.2 Synthesis of model peptide (3)

The synthesis was carried out according to the following scheme:

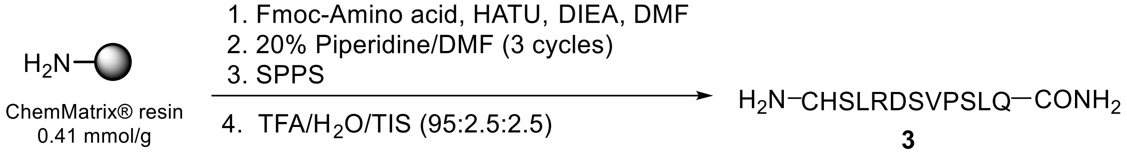

The synthesis of the model peptide 3 was carried out using Fmoc-SPPS on Rink amide ChemMatrix® resin (250 mg, loading 0.41 mmol/g, 0.1 mmol scale). The resin was pre-swollen with DMF for 15 min and all amino acids were coupled manually with Fmoc-amino acid (5 equiv.), HATU (5 equiv.) in 1.25 mL DMF and 0.25 mL DIEA for 45 mins. After each coupling step, the resin was washed 3 times with DMF and submitted to Fmoc-deprotection using 4 mL 20% piperidine/DMF containing 0.05% FA for 1 min, then 4 min, and another 1 min. After coupling all the amino acids, the peptide bound resin was washed with DMF (10 mL x 3) and DCM (10 mL x 3) and was kept under vacuum to dry. Subsequently a mixture of TFA/H_2O/TIS (95/2.5/2.5, 8 mL for 0.05 mmol scale) was added to the resin which was shaken for 2 h at room temperature. The resin was removed by filtration and washed with TFA (1 mL × 2). To precipitate the peptide, the combined filtrate was added dropwise to cold diethyl ether (35 mL for 0.025 mmol resin) followed by centrifugation at 4000 rpm for 4 min. Then, the ether was decanted, followed by dissolution of the peptide in 25% acetonitrile/water and lyophilized to get white powder. The crude dry peptide powder was purified by RP-HPLC (Method B that described in Section 1.3) affording the product 45.2 mg (yield 67%).

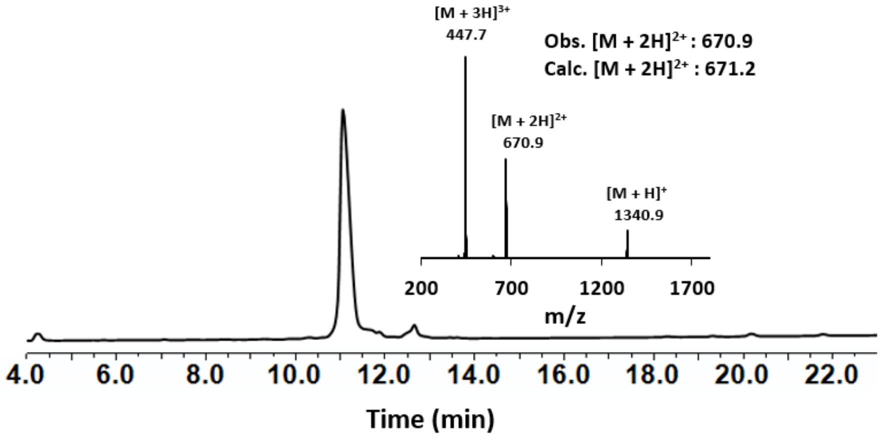

Figure S2. HPLC and MS analysis of model peptide (3): HPLC chromatogram of the UV absorbance at 214 nm. HPLC analysis was carried out with Method B (see section 1.2). Mass-to-charge (m/z) spectrum.

4.3 Synthesis of model peptide (4)

The synthesis was carried out according to the following scheme:

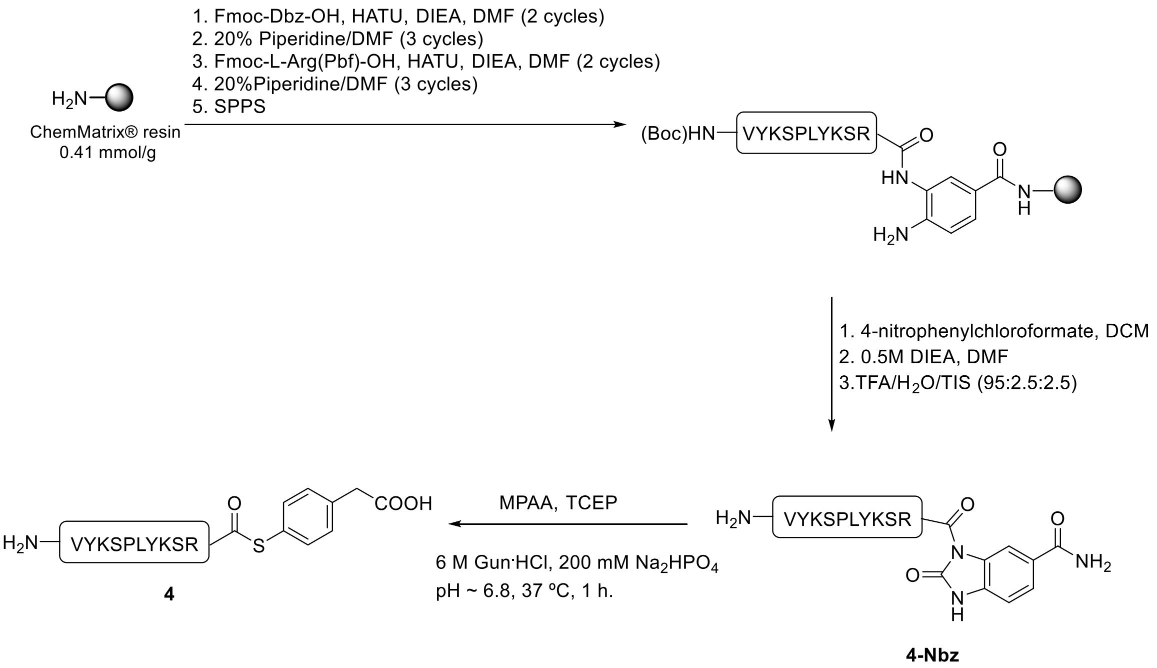

The synthesis of the model peptide 4 was carried out using Rink amide ChemMatrix® resin (250 mg, loading 0.41 mmol/g, 0.1 mmol scale). The resin was pre-swollen with DMF for 15 min and coupled with Fmoc-Dbz-OH (5 equiv.), using HATU (5 equiv.) and 0.25 mL DIEA in 1.25 mL DMF for 45 min (2 cycles). After deprotection with 20% piperidine/DMF containing 0.05% FA. The first amino acid was double coupled with Fmoc-L-Arg(Pbf)-OH (5 equiv.), using HATU (5 equiv.) and 0.25 mL DIEA in 1.25 mL DMF for 45 min (2 cycles). The remaining amino acids were coupled with the remaining sequence via Fmoc-amino acids (5 equiv.), using HATU (5 equiv.) and 0.25 mL DIEA in 1.25 mL DMF corresponding to the initial loading of the resin for 45 min. The last Amino acid was coupled with Boc-L-Val-OH (5 equiv.) using HATU (5 equiv.) and 0.25 mL DIEA in 1.25 mL for 45 min. The peptide bound resin was washed with DMF (5 mL x 3) and DCM (5 mL x 3) before treated with a solution of 5 equiv. of 4-nitrophenylchloroformate in 1.5 mL of DCM for 30 min (3 cycles). The resin was washed with DCM (5 mL x 3) and DMF (5 mL x 3), treated with a solution of 0.5 M DIEA in DMF and was shaken for 10 min (3 cycles) at room temperature. After that, the resin was washed with DMF (10 mL x 3) and DCM (10 mL x 3) and kept to dry under vacuum. Subsequently, a mixture of TFA/H_2O/TIS (95/2.5/2.5, 4 mL for 0.025 mmol) was added to the resin and was shaken for 2 h at room temperature. The resin was removed by filtration and washed with additional TFA (1 mL x 2). To precipitate the peptide, the combined filtrate was added dropwise to cold diethyl ether (35 mL for 0.025 mmol resin) followed by centrifugation at 4000 rpm for 4 min. Then the diethyl ether was decanted, followed by dissolution of the peptide in 25%

acetonitrile/water and lyophilized to get white powder. The crude dry peptide powder was purified by RP-HPLC (Method B that described in Section 1.3) affording the product 20.2 mg, yield 58%.

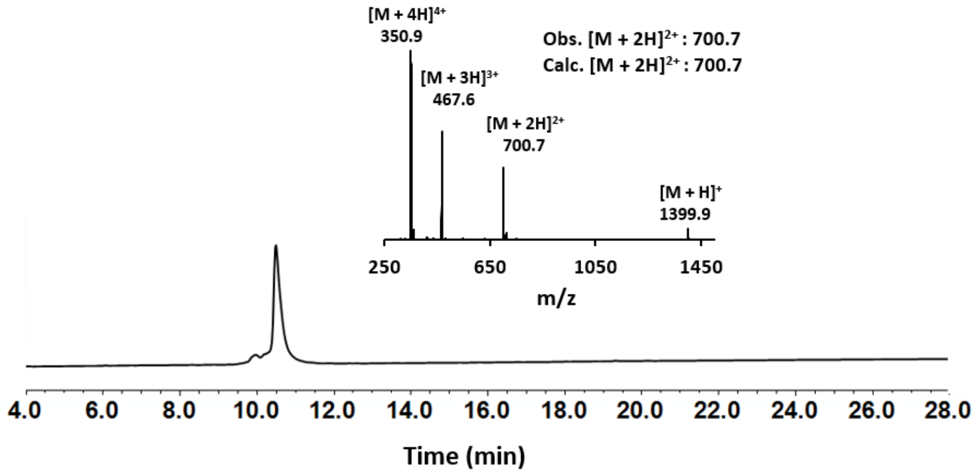

Figure S3. HPLC and MS analysis of model peptide (4)-Nbz: HPLC chromatogram of the UV absorbance at 214 nm. HPLC analysis of purified model peptide (4) was carried out with Method B (see section 1.2). Mass-to-charge (m/z) spectrum.

20.0 mg (4)-Nbz fragment was dissolved in 3.6 mL 6 M Gun·HCl, 200 mM Na_2HPO_4 buffer containing MPAA (30 equiv.) and TCEP (15 equiv.) at pH ~ 6.8 and incubated at 37 ºC for 1 h. The reaction was monitored by analytical RP-HPLC using C4 column and Method B (as described in Section 1.2). For preparative RP-HPLC, a gradient of 5-60% B over 60 min (Method B, as described in section1.3) was used to afford the corresponding peptide 7.2 mg as white powder (yield 36 %).

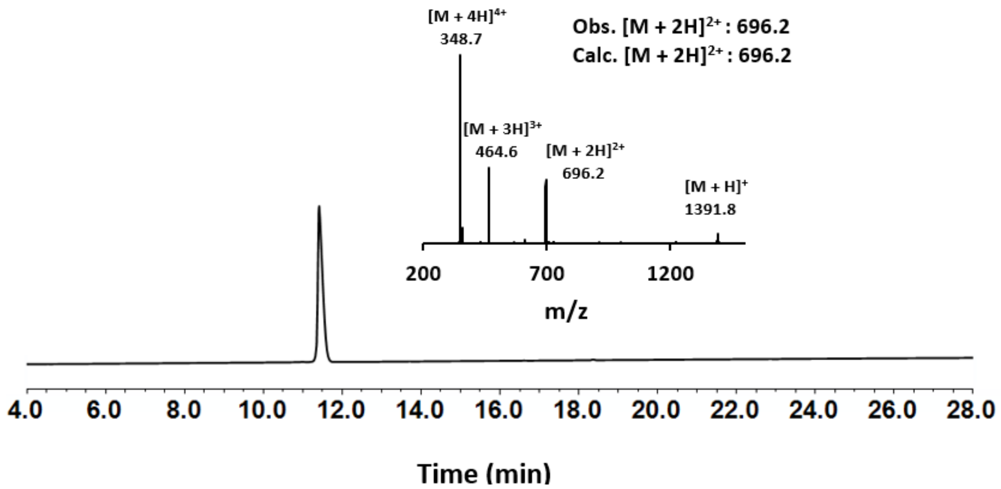

Figure S4. HPLC and MS analysis of model peptide (4). HPLC chromatogram of the UV absorbance at 214 nm. HPLC analysis was carried out with Method C (see section 1.2). Mass-to-charge (m/z) spectrum.

4.4 Max(22-42)-Nbz fragment (7)

The synthesis was carried out according to the following scheme:

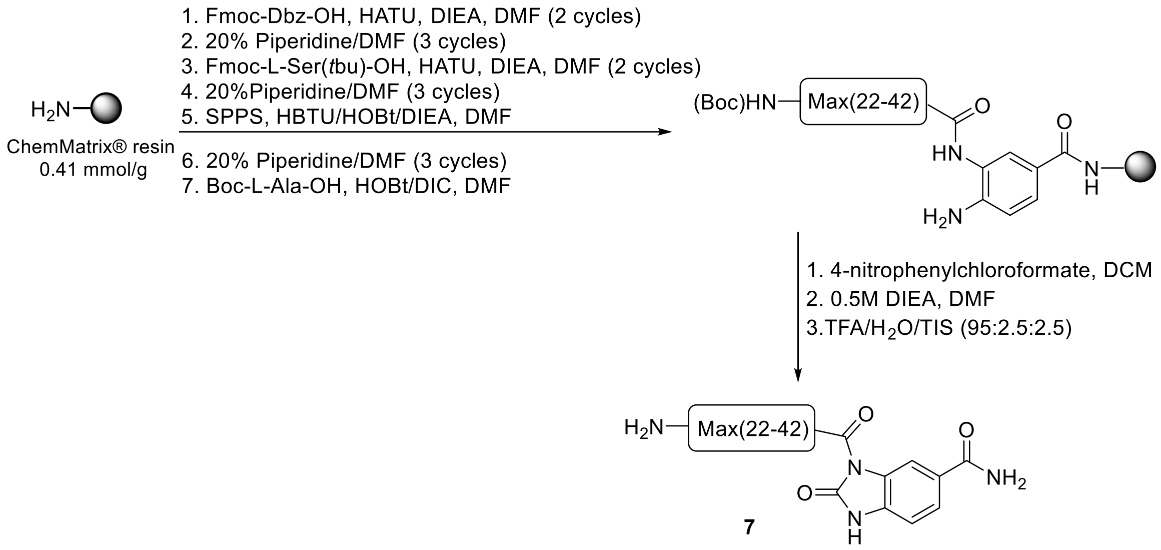

The synthesis of the model peptide 7 was carried out using Rink amide ChemMatrix® resin (250 mg, loading 0.41 mmol/g, 0.1 mmol scale). The resin was pre-swollen with DMF for 15 min and coupled with Fmoc-Dbz-OH (5 equiv.), using HATU (5 equiv.) and 0.25 mL DIEA in 1.25 mL DMF for 45 min (2 cycles). After deprotection with 20% piperidine, the first amino acid was double coupled with Fmoc-L-Ser(*t*bu)-OH (5 equiv.) using HATU (5 equiv.) and 0.25 mL DIEA in 1.25 mL DMF for 45 min (2 cycles). The remaining amino acids were coupled with the remaining sequence via Fmoc-amino acids (5 equiv.), using HBTU/HOBt (5 equiv./ 5 equiv.) corresponding to the initial loading of the resin and 174 μl DIEA in 1.25 mL DMF for 45 min. The last Amino acid was coupled with Boc-L-Ala-OH (5 equiv.) using HOBt/DIC (5 equiv./ 5 equiv.) for 1h. The peptide bound resin was washed with DMF and DCM before treated with a solution of 4-nitrophenylchloroformate (5 equiv.) in 1.5 mL DCM for 30 min (3 cycles). The resin was washed with DCM and DMF and treated with a solution of 0.5 M DIEA in DMF and was shaken for 10 min (3 cycles) at room temperature. After that, the resin was washed with DMF (5 mL x 3) and DCM (5 mL x 3) and kept to dry under vacuum. Subsequently, a mixture of TFA/H_2O/TIS (95/2.5/2.5, 4 mL for 0.025 mmol) was added to the resin and was shaken for 2 h at room temperature. The resin was removed by filtration and washed with additional TFA (2 × 1 mL). To precipitate the peptide, the combined filtrate was added dropwise to cold diethyl ether (35 mL for 0.025 mmol resin) followed by centrifugation at 4000 rpm for 4 min. Then the diethyl ether was decanted, followed by dissolution of the peptide in 25% acetonitrile/water and lyophilized to get white powder. The crude dry peptide powder was purified by RP-HPLC (Method B as described in Section 1.3) affording the product 39.8 mg as a white powder (yield 59%).

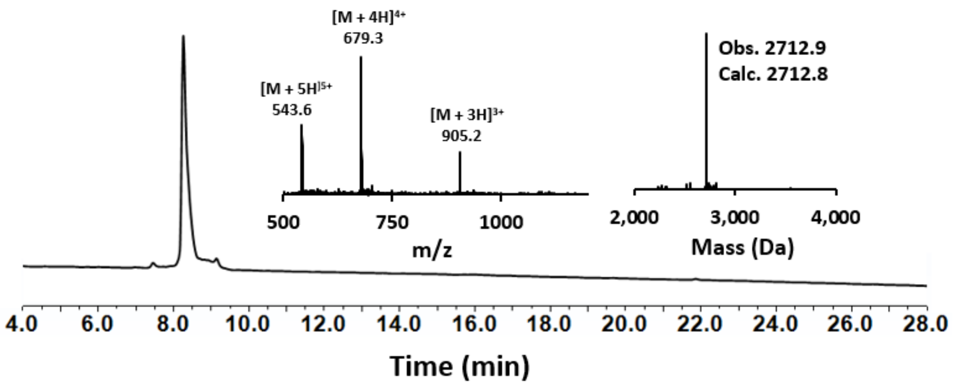

Figure S5. HPLC and MS analysis of Max(22-42)-Nbz fragment (7). HPLC chromatogram of the UV absorbance at 214 nm. HPLC analysis was carried out with Method B (see section 1.2). Mass-to-charge (m/z) spectrum, and deconvoluted mass spectrum.

4.5 Cys-Max(44-102) fragment (8)

The synthesis was carried out according to the following scheme:

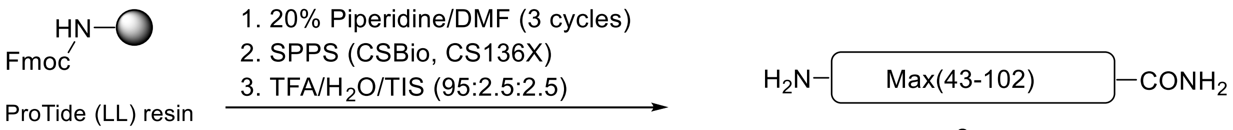

8

The synthesis of Cys-Max(44-102) 8 was carried out on Rink amide ProTide (LL) resin (0.18 mmol/g, 0.1 mmol scale) by CSBio (CS136X). Resin was pre-swollen in DMF (5 mL) for 15 min, 20% piperidine/DMF (5 min x 3) and then loaded into the reaction vessel (20 mL). The standard synthetic cycle involves: deprotection (20% piperidine/DMF), wash, followed by coupling with Fmoc-L-amino acid (5 equiv. in 5 mL DMF) at room temperature using HBTU/HOBt (5 equiv./5 equiv.) and 0.35 mL DIEA in 4.65 mL DMF. The deprotection–wash-coupling cycle is repeated for all additional monomers. The Last amino acid Boc-L-Cys(Trt)-OH was coupled manually using HOBt/DIC (5 equiv./ 5 equiv.) for 60 min at room temperature. Then, the resin was washed 3 times with DMF (10 mL x 3) and DCM (10 mL x 3) and kept to dry under vacuum. A Mixture of TFA/H_2O/TIS (95/2.5/2.5, 6 mL for 0.025 mmol) was added to the resin which was shaken for 3 h at room temperature. The resin was removed by filtration and was washed with additional TFA (1 mL × 2). To precipitate the peptide, the combined filtrate was added dropwise to cold diethyl ether (35 mL for 0.025 mmol resin) followed by centrifugation at 4000 rpm for 4 min. Subsequently, the diethyl ether was decanted, followed by dissolution of the peptide in 25% acetonitrile/water and lyophilized to get white powder. The crude dry peptide powder was purified by RP-HPLC (Method B and Method C that described in Section 1.3) affording the pure product 27.6 mg

as white powder (yield 16%). *Note: After purification by Method B (Section 1.3), the semi-pure peptide was collected and purified by Method C (Section 1.3) to get pure peptide fragment.*

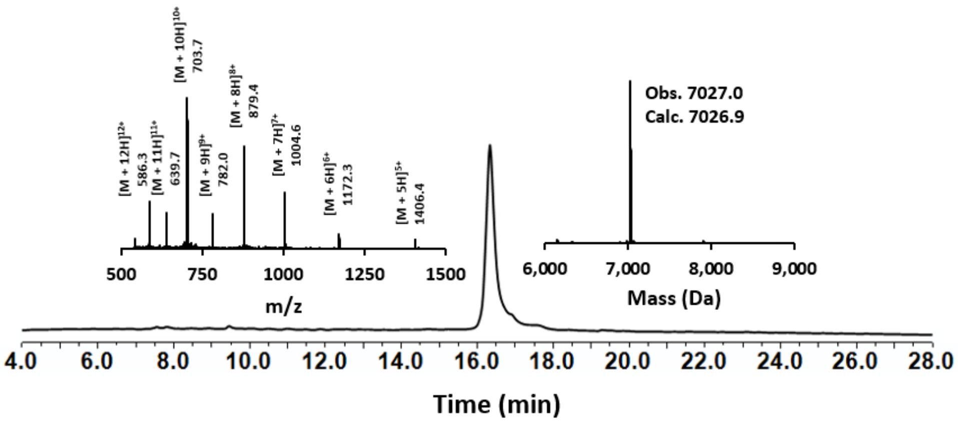

Figure S6. HPLC and MS analysis of Cys-Max(44-102) fragment (8). HPLC chromatogram of the UV absorbance at 214 nm. HPLC analysis was carried out with Method B (see section 1.2). Mass-to-charge (m/z) spectrum, and deconvoluted mass spectrum

4.6 Cys-Myc(403-434) fragment (9)

The synthesis was carried out according to the following scheme:

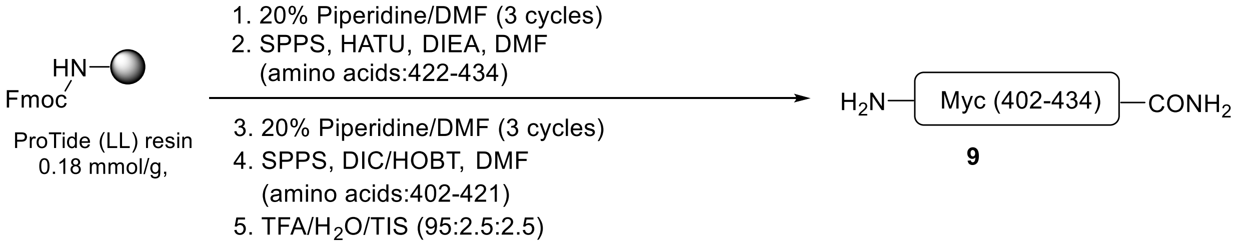

The synthesis of Cys-Myc(403-434) 9 was carried out on Rink amide ProTide (LL) resin (loading 0.18 mmol/g, 0.1 mmol scale). The resin was pre-swollen in DMF (5 mL) for 15 min and then treated with 5 mL 20% piperidine (5 min x 3), followed by washing with DMF (10 mL x 3). For the first amino acid Leu434 to Lys422, the resin was coupled with Fmoc-amino acid (5 equiv.) using HATU (5 equiv.) and 0.25 mL DIEA in 1.25 mL DMF for 30 min. For the Arg421 to Tyr402, the resin was coupled with Fmoc-amino acid (5 equiv.) using HOBt/DIC (5 equiv./ 5 equiv.) for 60 min. After coupled all amino acids, the peptide bound resin was washed with DMF (10 mL x 3) and DCM (10 mL x 3) and kept to dry under vacuum. Subsequently, a mixture of TFA/H_2O/TIS (95/2.5/2.5, 6 mL for 0.025 mmol scale) was added to the resin which was shaken for 2 h at room temperature. The resin was removed by filtration and was washed with additional TFA (1 mL x 2). To precipitate the peptide, the combined filtrate was added dropwise to cold diethyl ether (35 mL for 0.025 mmol resin) followed by centrifugation at 4000 rpm for 4 min. Then the diethyl ether was decanted, followed by dissolution of the peptide in 25% acetonitrile/water

and lyophilized to get white powder. The crude dry peptide powder was purified by RP-HPLC (Method B that described in Section 1.3) affording the product 32.9 mg product as white powder (yield 32%).

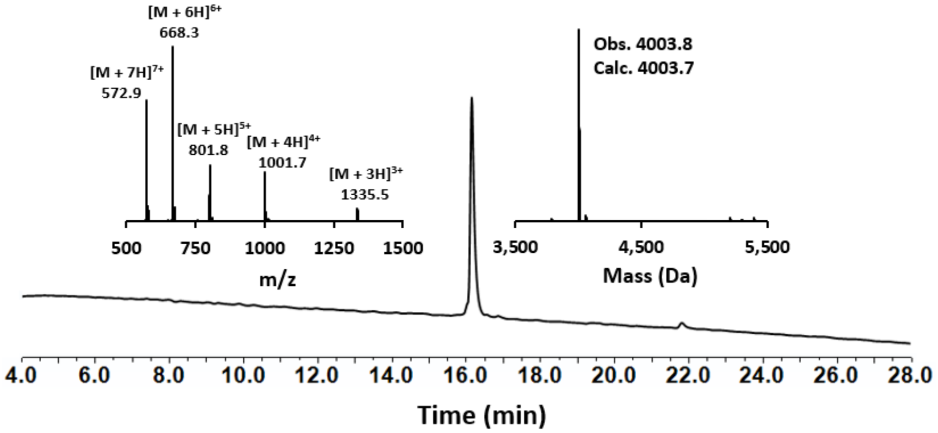

Figure S7. HPLC and MS analysis of Cys-Myc(403-434) fragment (9). HPLC chromatogram of the UV absorbance at 214 nm. HPLC analysis was carried out with Method B (see section 1.2). Mass-to-charge (m/z) spectrum, and deconvoluted mass spectrum.

4.7 Myc(354-401)-Nbz fragment (10)

The synthesis was carried out according to the following scheme:

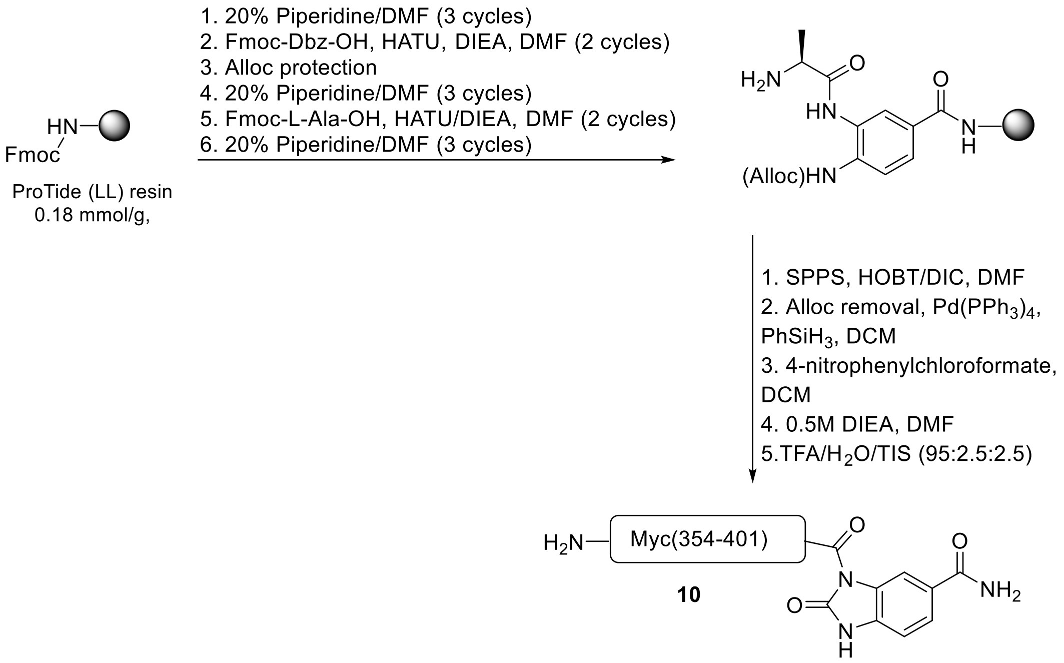

The synthesis of Myc(354-401)-Nbz 10 was carried out using Rink amide ProTide (LL) (0.18 mmol/g, 0.1 mmol scale). The resin was pre-swollen in DMF (5 mL) for 15 min, treated with 5 mL 20% piperidine/DMF (5 min x 3), washed with DMF (10 mL x 3) and coupled with Fmoc-Dbz-OH (5 equiv.),

using HATU (5 equiv) and 0.25 mL DIEA for 45 min (2 cycle). Subsequently, the resin was washed with DMF (5 mL x 3) and DCM (5 mL x 3) and was treated with a solution of Allyl chloroformate (50 equiv.) and DIEA (2 equiv.) in DCM for 12 h at room temperature. Following the Fmoc removal, the first amino acid (Fmoc-L-Ala-OH) was manually double coupled using HATU (5 equiv.) and 0.25 mL DIEA for 45 min (2 cycle). The remaining sequence was coupled with Fmoc-amino acid (5 equiv.), using HOBt/DIC (5 equiv./ 5 equiv.) for 60 mins. After that the peptide bound resin was washed with DMF (10 mL x 3) and DCM (10 mL x 3), and Alloc-protected resin was treated with a solution of $PhSiH_3$ (20 equiv.) and $Pd(PPh_3)_4$ (0.35 equiv.) in DCM and shaken for 30 min at 25 °C. The resin was washed with DCM and a solution of 5 equiv. of 4-nitrophenylchloroformate in 1.5 mL of DCM. The resin was shaken for 30 min (3 cycles) at 25 °C and washed with DCM (5 mL x 3) and DMF (5 mL x 3). To the washed resin, a solution of 0.5 M DIEA in DMF was added and shaken for additional 10 min (3 cycles) to complete the cyclization. The resin was washed with DMF (5 mL x 3), DCM (5 mL x 3) and kept to dry under vacuum. Subsequently, a mixture of TFA/H_2O/TIS (95/2.5/2.5, 8 mL for 0.05 mmol scale) was added to the resin which was shaken for 2 h at room temperature. The resin was removed by filtration and was washed with additional TFA (1 mL × 2). To precipitate the peptide, the combined filtrate was added dropwise to cold diethyl ether (35 mL for 0.025 mmol resin) followed by centrifugation at 4000 rpm for 4 min. Then, the ether was decanted, followed by dissolution of the peptide in 25% acetonitrile/water and lyophilized to get white powder. The crude dry peptide powder was purified by RP-HPLC (Method B that described in Section 1.3) affording 27.0 mg product as white powder (10% yield).

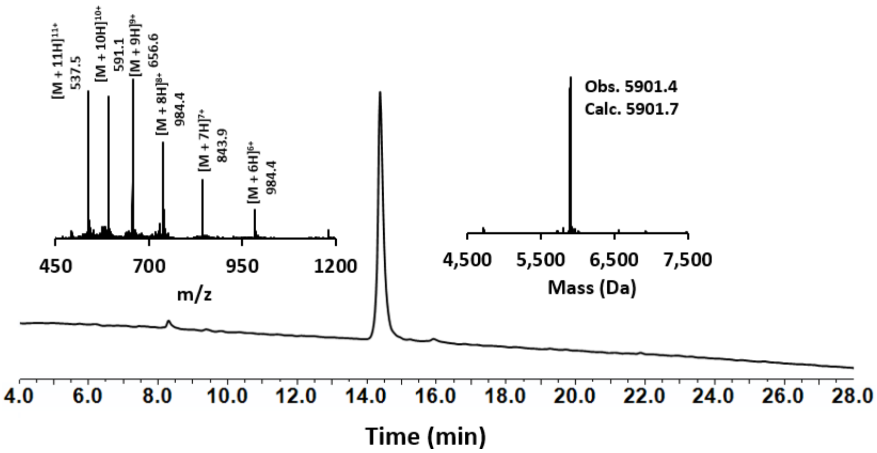

Figure S8. HPLC and MS analysis of Myc(354-401)-Nbz fragment (10). HPLC chromatogram of the UV absorbance at 214 nm. HPLC analysis was carried out with Method B (see section 1.2). Mass-to-charge (m/z) spectrum, and deconvoluted mass spectrum.

5. S-arylation of peptide P1

5.1 Evaluation of Reaction Conditions for Peptide Arylation

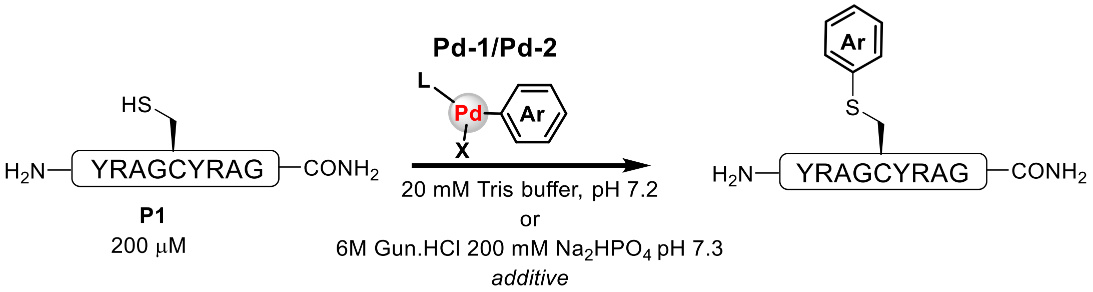

General procedure of the model peptide P1 arylation with Pd-1 and Pd-2

1. P1 with Pd-1 in Tris buffer (Entry 1):

To a 1.5 mL Eppendorf tube was added P1 (50 µL, 1 mM, 1.0 equiv.) as a solution in 20 mM Tris (pH 7.2), 20 mM Tris (175 µL, pH 7.2), and Pd-1 (25 µL, 10 mM, 5 equiv.) as a solution in DMF. The final reaction concentrations of the major reaction components were the following: P1 (0.2 mM); Pd-1 (1 mM). The Eppendorf tube was then closed and the reaction mixture was vortexed and kept at room temperature for 15 min. Next, the Eppendorf tube was opened and the reaction was treated with 3-MPA (10 equiv. compared to Pd-1) and kept at room temperature for 5 min. 20 µL of reaction mixture was injected to Analytical HPLC using Method A (Section 1.2) and Mass spectrum was acquired using Thermo Scientific ISQ EM Mass spectrometer. Result was depicted in Figure S9-b and table Entry 1.

2. P1 with Pd-1 in 6 M Gun·HCl buffer (Entry 2):

To a 1.5 mL Eppendorf tube was added P1 (50 µL, 1 mM, 1.0 equiv.) as a solution in 6 M Gun·HCl 200 mM Na_2HPO_4 (pH 7.3), 6 M Gun·HCl 200 mM Na_2HPO_4 (175 µL, pH 7.3), and Pd-1 (25 µL, 10 mM, 5 equiv.) as a solution in DMF. The final reaction concentrations of the major reaction components were the following: P1 (0.2 mM); Pd-1 (1 mM). The Eppendorf tube was then closed and the reaction mixture was vortexed and kept at room temperature for 15 min. Next, the Eppendorf tube was opened and the reaction was treated with 3-MPA (10 equiv. compared to Pd-1) and kept at room temperature for 5 min. 20 µL of reaction mixture was injected to Analytical HPLC using Method A (see section 1.2) and Mass spectrum was acquired using Thermo Scientific ISQ EM Mass spectrometer. Result was depicted in Figure S9-c and table Entry 2.

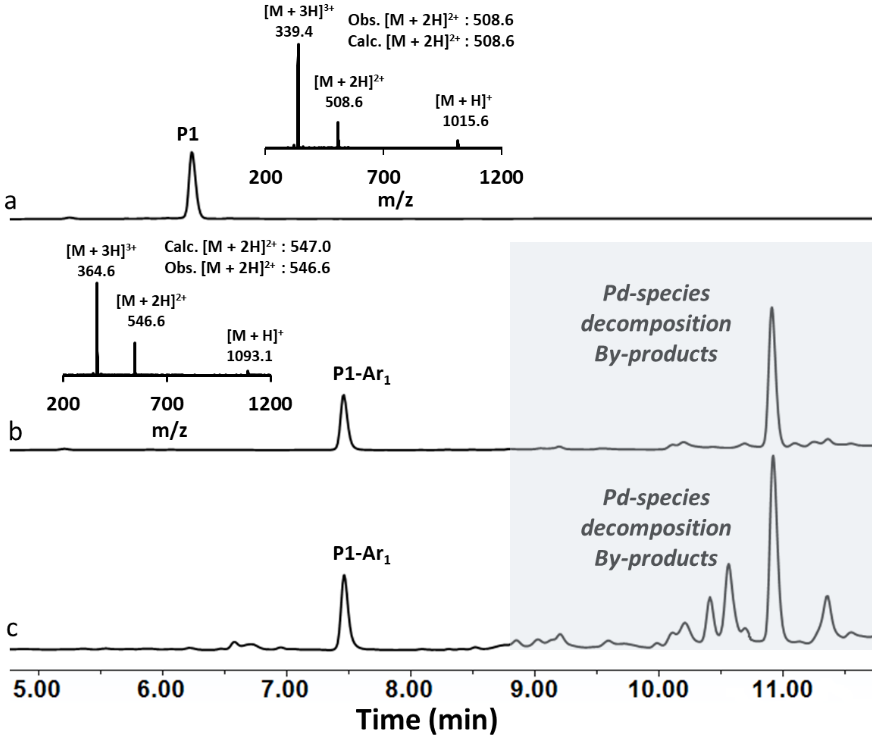

Figure S9. HPLC and MS analysis of the S-arylation of peptide P1 via Pd-1. HPLC chromatogram of the UV absorbance at 214 nm. HPLC analysis was carried out with Method A (see section 1.2). Mass-to-charge (m/z) spectrum. (a) peptide P1 at t = 0. (b) crude reaction of P1 with Pd-1 in 20 mM Tris buffer after 15 min. (c) crude reaction of P1 with Pd-1 in 6 M Gun·HCl 200 mM Na_2HPO_4 buffer after 15 min.

3. P1 with Pd-2 in Tris buffer (Entry 3):

To a 1.5 mL Eppendorf tube was added P1 (50 µL, 1 mM, 1.0 equiv.) as a solution in 20 mM Tris (pH 7.2), 20 mM Tris (175 µL, pH 7.2), and Pd-2 (25 µL, 10 mM, 5 equiv.) as a solution in DMF. The final reaction concentrations of the major reaction components were the following: P1 (0.2 mM); Pd-2 (1 mM). The Eppendorf tube was then closed and the reaction mixture was vortexed and kept at room temperature for 15 min. Next, the Eppendorf tube was opened and the reaction was treated with 3-MPA (10 equiv. compared to Pd-2) and kept at room temperature for 5 min. 20 µL of reaction mixture was injected to Analytical HPLC using Method A (see Section 1.2) and Mass spectrum was acquired using Thermo Scientific ISQ EM Mass spectrometer. Result was depicted in Figure S10-b and table Entry 3.

4. P1 with Pd-2 in 6 M Gun·HCl buffer (Entry 4):

To a 1.5 mL Eppendorf tube was added P1 (50 µL, 1 mM, 1.0 equiv.) as a solution in 6 M Gun·HCl 200 mM Na_2HPO_4, 6 M Gun·HCl 200 mM Na_2PO_4 (175 µL, pH 7.3), and Pd-2 (25 µL, 10 mM, 5 equiv.) as a solution in DMF. The final reaction concentrations of the major reaction components were the following: P1 (0.2 mM); Pd-2 (1 mM). The Eppendorf tube was then closed, and the reaction mixture was vortexed and kept at room temperature for 15 min. Next, the Eppendorf tube was opened, and the reaction was treated with 3-MPA (10 equiv. compared to Pd-2) and kept at room temperature for 5 min. 20 µL of reaction mixture was injected to Analytical HPLC using Method A (see Section 1.2) and Mass spectrum was acquired using Thermo Scientific ISQ EM Mass spectrometer. Result was depicted in Figure S10-c and table Entry 4.

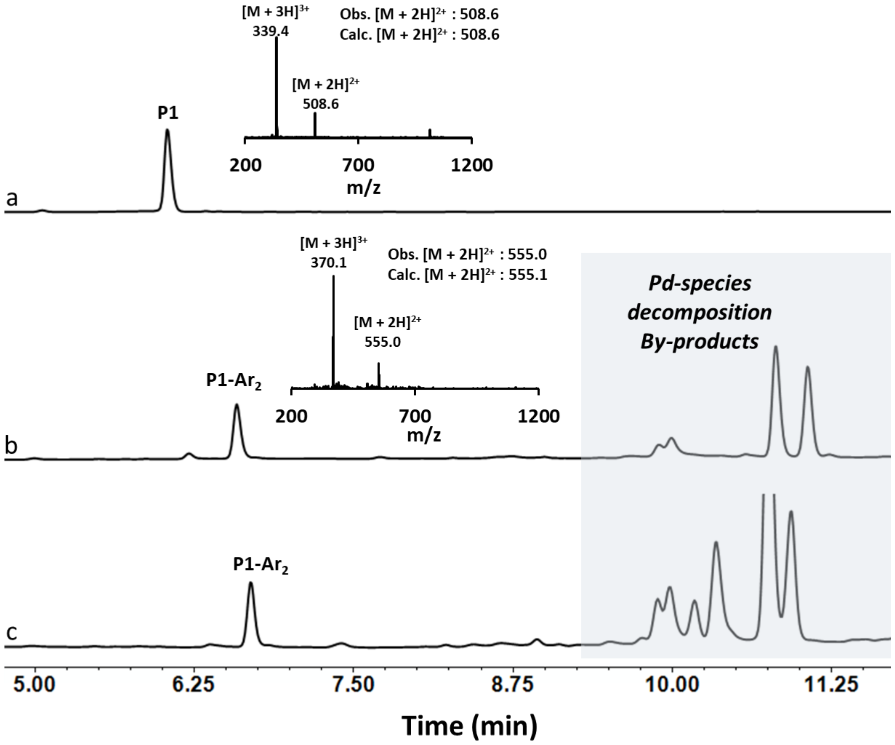

Figure S10. HPLC and MS analysis of the S-arylation of peptide P1 via Pd-2. HPLC chromatogram of the UV absorbance at 214 nm. HPLC analysis was carried out with Method A (see section 1.2). Mass-to-charge (m/z) spectrum. (a) peptide P1 at t = 0. (b) crude reaction of P1 with Pd-2 in 20 mM Tris buffer after 15 min. (c) crude reaction of P1 with Pd-2 in 6 M Gun·HCl 200 mM Na_2HPO_4 buffer after 15 min.

5. P1 with Pd-1 in the presence of TCEP additive (Entry 5):

To a 1.5 mL Eppendorf tube was added P1 (50 µL, 1 mM, 1.0 equiv.) as a solution in in 6 M Gun·HCl 200 mM Na_2HPO_4, (pH 7.3), 6 M Gun·HCl 200 mM Na_2HPO_4 (175 µL, pH 7.3) containing TCEP (10 equiv.), and Pd-1 (25 µL, 10 mM, 5 equiv.) as a solution in DMF. The final reaction concentrations of the major reaction components were the following: P1 (0.2 mM); TCEP (2 mM); Pd-1 (1 mM). The Eppendorf tube was then closed and the reaction mixture was vortexed and kept at room temperature for 15 min. Next, the Eppendorf tube was opened, and the reaction was treated with 3-MPA (10 equiv. compared to Pd-1) and kept at room temperature for 5 min. 20 µL of reaction mixture was injected to Analytical HPLC using method A (see Section 1.2) and Mass spectrum was acquired using Thermo Scientific ISQ EM Mass spectrometer. Result was depicted in Figure S11-b and table Entry 5.

6. P1 with Pd-1 in the presence of TCEP additive (Entry 6):

To a 1.5 mL Eppendorf tube was added P1 (50 µL, 1 mM, 1.0 equiv.) as a solution in in 6 M Gun·HCl 200 mM Na_2HPO_4, (pH 7.3), 6 M Gun·HCl 200 mM Na_2HPO_4 (300 µL, pH 7.3) containing TCEP (10 equiv.), and Pd-1 (50 µL, 10 mM, 10 equiv.) as a solution in DMF. The final reaction concentrations of the major reaction components were the following: P1 (0.125 mM); TCEP (1.25 mM): Pd-1 (1.25 mM). The Eppendorf tube was then closed and the reaction mixture was vortexed and kept at room temperature for 15 min. Next, the Eppendorf tube was opened and the reaction was treated with 3-MPA (10 equiv. compared to Pd-1) and kept at room temperature for 5 min. 20 µL of reaction mixture was injected to Analytical HPLC using method A (see Section 1.2) and Mass spectrum was acquired using Thermo Scientific ISQ EM Mass spectrometer. Result was depicted in Figure S11-c and table Entry 6.

6. P1 with Pd-1 in the presence of MPAA additive (Entry 7):

To a 1.5 mL Eppendorf tube was added P1 (50 µL, 1 mM, 1.0 equiv.) as a solution in 6 M Gun·HCl 200 mM Na_2HPO_4, (pH 7.3), 6 M Gun·HCl 200 mM Na_2HPO_4 (175 µL, pH 7.3) containing MPAA (15 equiv.), and Pd-1 (25 µL, 10 mM, 5 equiv.) as a solution in DMF. The final reaction concentrations of the major reaction components were the following: P1 (0.2 mM); MPAA (3 mM); Pd-1 (1 mM). The Eppendorf tube was then closed and the reaction mixture was vortexed and kept at room temperature for 15 min. 20 µL of reaction mixture was injected to Analytical HPLC using method A (see Section 1.2) and Mass spectrum was acquired using Thermo Scientific ISQ EM Mass spectrometer. Result was depicted in Figure S11-d and table Entry 7.

7. P1 with Pd-1 in the presence of TCEP and MPAA additives (Entry 8):

To a 1.5 mL Eppendorf tube was added P1 (50 µL, 1 mM 1.0 equiv.) as a solution in 6 M Gun·HCl 200 mM Na_2PO_4, 6 M Gun·HCl 200 mM Na_2PO_4 (150 µL, pH 7.3) containing TCEP (10 equiv.) and MPAA (15 equiv.), and Pd-1 (50 µL, 10 mM, 20 equiv.) as a solution in DMF. The final reaction concentrations of the major reaction components were the following: P1 (0.2 mM); TCEP (2 mM); MPAA (3 mM); Pd-1 (4 mM). The Eppendorf tube was then closed and the reaction mixture was vortexed and kept at room temperature for 15 min. Next, the Eppendorf tube was opened and the reaction was treated with 3-MPA (10 equiv. compared to Pd-1) and kept at room temperature for 5 min. 20 µL of reaction mixture was injected to Analytical HPLC using method A (see Section 1.2) and Mass spectrum was acquired using Thermo Scientific ISQ EM Mass spectrometer. Result was depicted in Figure S11-e and table Entry 8.

8. P1 with Pd-1 in Imidazole buffer (Entry 9)

To a 1.5 mL Eppendorf tube was added P1 (50 µL, 1 mM 1.0 equiv.) as a solution in 5 M Imidazole, 5 M Imidazole (325 µL, pH 7.0), and Pd-1 (25 µL, 20 mM, 10 equiv.) as a solution in DMF. The final reaction concentrations of the major reaction components were the following: P1 (0.125 mM); Pd-1 (1.25 mM). The Eppendorf tube was then closed and the reaction mixture was vortexed and kept at 37 °C for 40 min. Next, the Eppendorf tube was opened and the reaction was treated with 3-MPA (10 equiv. compared to Pd-1) and kept at room temperature for 5 min. 20 µL of reaction mixture was injected to Analytical HPLC using method A (see Section 1.2) and Mass spectrum was acquired using Thermo Scientific ISQ EM Mass spectrometer. Result was depicted in Figure S11-f and table Entry 9.

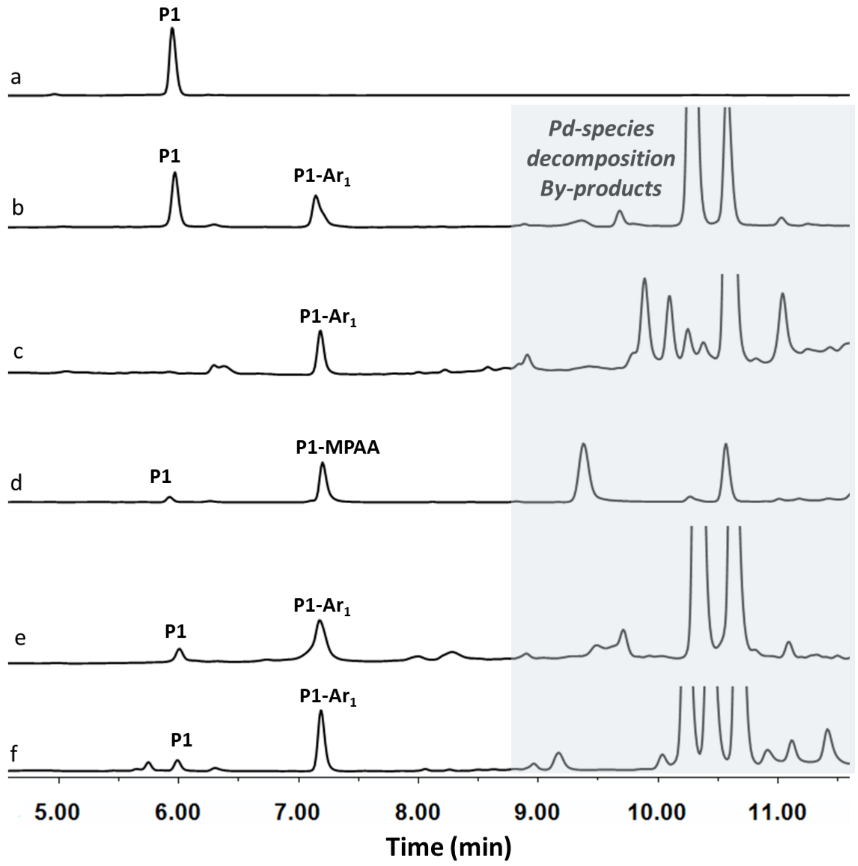

Figure S11. HPLC and MS analysis of the S-arylation of peptide P1 via Pd-1 in the presence of additives. HPLC chromatogram of the UV absorbance at 214 nm. HPLC analysis was carried out with Method A (see section 1.2). Mass-to-charge (m/z) spectrum. (a) peptide P1 at t = 0. (b) crude reaction of P1 with Pd-1 (5 equiv.) in the presence of TCEP. (c) crude reaction of P1 with Pd-1 (10 equiv.) in the presence of TCEP. (d) crude reaction of P1 with Pd-1 (5 equiv.) in the presence of MPAA. (e) crude reaction of P1 with Pd-1. (20 equiv.) in the presence of TCEP/MPAA. (f) crude reaction of P1 with Pd-1 (10 equiv.) in 5 M Imidazole buffer.

**Entry**	**Buffer**	**Pd complex**	**Additive**	**Yield**
**1**	20 mM Tis (pH 7.2)	Pd-1<br>5 equiv.	—	>99%
**2**	6 M Gun·HCl 200 mM phosphate buffer<br>(pH 7.3)	Pd-1<br>5 equiv.	—	>99%
**3**	20 mM Tis (pH 7.2)	Pd-2<br>5 equiv.	—	>99%
**4**	6 M Gun·HCl 200 mM phosphate buffer<br>(pH 7.3)	Pd-2<br>5 equiv.	—	>99%
**5**	6 M Gun·HCl 200 mM phosphate buffer<br>(pH 7.3)	Pd-1<br>5 equiv.	TCEP<br>(10 equiv.)	44%
**6**	6 M Gun·HCl 200 mM phosphate buffer<br>(pH 7.3)	Pd-1<br>10 equiv.	TCEP<br>(10 equiv.)	>99%
**7**	6 M Gun·HCl 200 mM phosphate buffer<br>(pH 7.3)	Pd-1<br>5 equiv.	MPAA<br>(15 equiv.)	(90%)[a]
**8**	6 M Gun·HCl 200 mM phosphate buffer<br>(pH 7.3)	Pd-1<br>20 equiv.	MPAA/TCEP<br>(15equiv./10equiv.)	88%
**9**	5 M Imidazole<br>(pH 7.0)	Pd-1<br>10 equiv.	—	89%

[a] in Entry 7, oxidized P1-MPPA.

6. Native Chemical Ligation and One-Pot S-arylation with PdOACs

6.1 One-pot NCL and S-arylation

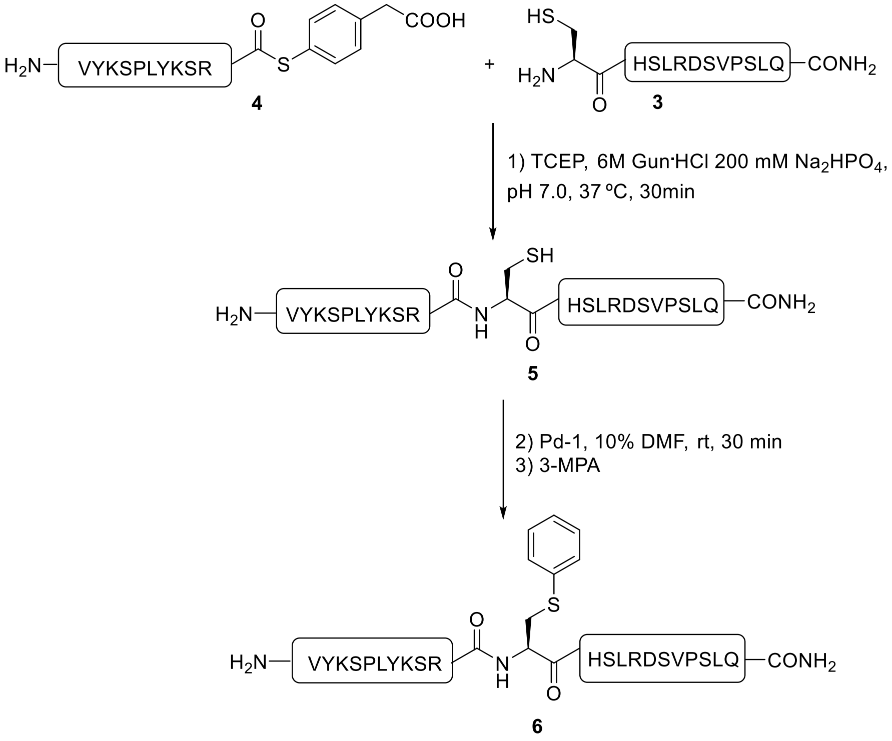

Peptide 3 (1.3 mg, 1.0 µmol, 1 equiv.) and peptide 4 (1.7 mg, 1.2 µmol 1.2 equiv.) were dissolved in 6 M Gun·HCl, 200 mM Na_2HPO_4 buffer (485 µL, 2 mM) containing TCEP (10 mM) at pH 7.0. The mixture was incubated at 37 ºC for 30 min. The reaction was followed using an analytical C4 column and Method C (see Section 1.2). After the reaction was finished, the ligation solution was diluted to 500 µM using 6 M Gun·HCl, 200 mM Na_2HPO_4 buffer pH 7.3, and Pd-1 in DMF (8.0 mg, 10 µmol, 10 equiv., V_{DMF}/V_{total} = 10 %) was added in one portion. Then, the reaction mixture was vortexed and left at room temperature for 30 mins. The process was monitored by analytical HPLC by Method C (see section 1.2). HPLC-MS analysis showed the formation of S-arylation peptide and consumption of the ligated peptide. 3-MPA (5 equiv. compared to Pd-1) was added to quench reaction. After an additional 10 min, S-arylation peptide was purified by RP-HPLC (Method B as described in section 1.3) to afford 1.5 mg as white powder (57% yield).

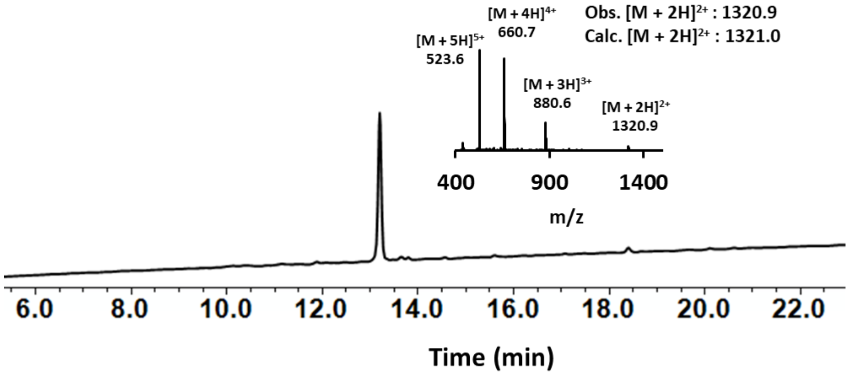

Figure S12. HPLC and MS analysis of isolated 6. HPLC chromatogram of the UV absorbance at 214 nm. HPLC analysis was carried out with Method B (see section 1.2). Mass-to-charge (m/z) spectrum.

6.2 Total chemical synthesis of Max via NCL and one-pot S-arylation

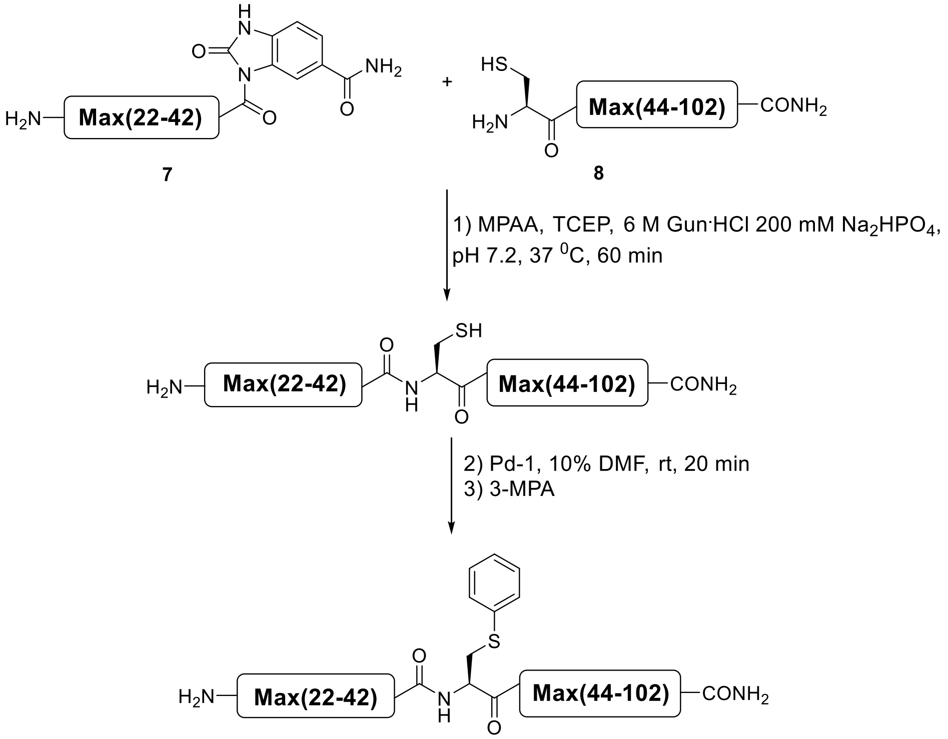

Cys-Max(44-102) fragment 8 (4.0 mg, 0.57 µmol, 1 equiv.) and Max(22-42)-Nbz fragment 7 (2.3 mg, 0.85 µmol, 1.5 equiv.) were dissolved in 6 M Gun·HCl, 200 mM Na_2HPO_4 buffer (284.6 µL, 2mM) containing 30 equiv. of MPAA and 15 equiv. of TCEP at pH ~7.2. The reaction mixture was incubated at 37 °C for 60 min. The progress of the reaction was monitored by analytical HPLC by Method B (Section 1.2). After completion of the reaction, the crude reaction was desalted by pipetting the reaction mixture into a 3 kDa molecular weight cutoff spin filter (Amicon® Ultra- 2mL, 3K). The reaction mixture was diluted to 2.0 mL using 20 mM Tris buffer (pH 7.2) and concentrated to 1.0 mL at 5000 rpm for 15 min.

This process was repeated three times. After that, reaction mixture was collected by reverse centrifuge. The reaction mixture was diluted to 250 µM, followed by adding Pd-1 which was dissolved in DMF (2.3 mg, 5 equiv., (v_{DMF}/v_{total} = 10%)). The reaction was kept at room temperature for 20 min and analyzed by analytical HPLC (C4 column) using method B (section 1.2). 3-MPA (5 equiv. compared with Pd-1 complex) was added for an additional 10 min at room temperature to quench reaction. Finally, modified Max was purified by RP-HPLC (Method B as described in section 1.3) to afford 1.4 mg as white powder (25% yield).

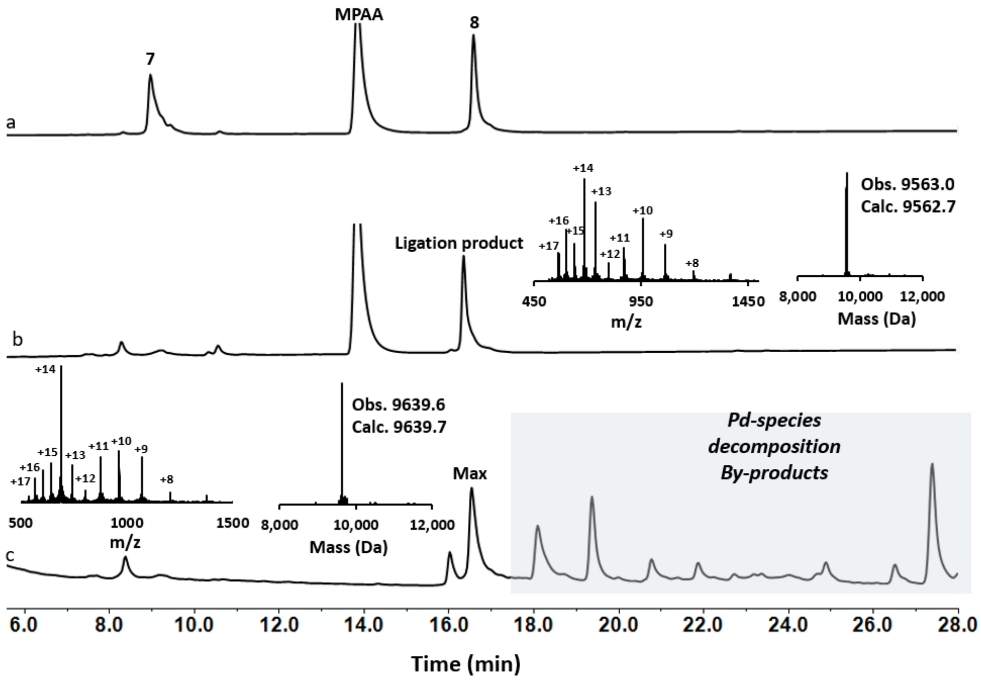

Figure S13. HPLC and MS analysis of the chemical synthesis of Max. HPLC chromatogram of the UV absorbance at 214 nm. HPLC analysis was carried out with Method B (see section 1.2). Mass-to-charge (m/z) spectrum, and deconvoluted mass spectrum. (a) Ligation at t = 0 for Max(22-42)-Nbz fragment 7 and Cys-Max(44-102) fragment 8. (b) Crude ligation reaction at t = 60 min. (c) Crude ligation and S-arylation reaction with Pd-1 at t = 20 min.

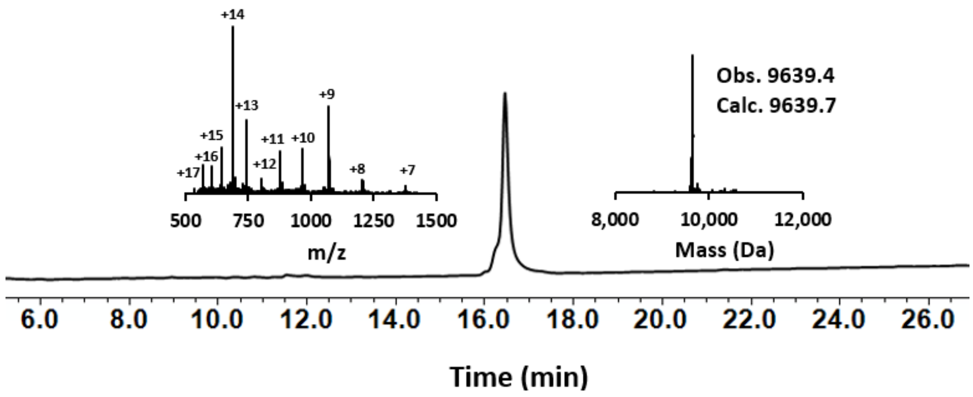

Figure S14. HPLC and MS analysis of isolated Max. HPLC chromatogram of the UV absorbance at 214 nm. HPLC analysis was carried out with Method B (see section 1.2). Mass-to-charge (m/z) spectrum, and deconvoluted mass spectrum.

6.3 Total chemical synthesis of Myc via NCL and one-pot S-arylation

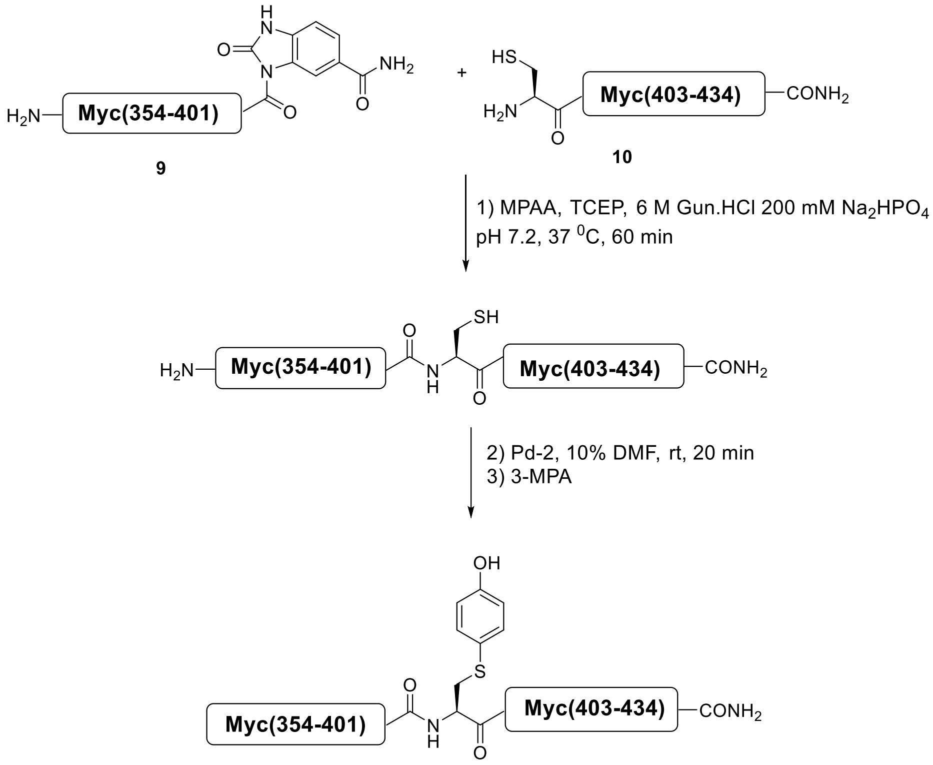

Cys-Myc(403-434) fragment 10 (2.3 mg, 0.57 µmmol, 1 equiv.) and Myc(354-401)-Nbz fragment 9 (4.1 mg, 0.69 µmol, 1.2 equiv.) were dissolved in 6 M Gun·HCl, 200 mM Na_2HPO_4 buffer (287 µL, 2mM) containing 30 equiv. of MPAA and 15 equiv. of TCEP at pH ~7.2. The reaction mixture was incubated at 37 °C for 60 min. The progress of the reaction was monitored by analytical HPLC by method B (see section 1.2). After completion of the reaction, the crude reaction was desalted by pipetting the reaction

mixture into a 3 kDa molecular weight cutoff spin filter (Amicon® Ultra- 2mL, 3K). The reaction mixture was diluted to 2.0 mL using 20 mM Tris buffer (pH 7.2) to and concentrated at 5000 rpm for 15 min. This process was repeated three times. The reaction mixture was diluted to 250 µM, followed by adding Pd-2 which was dissolved in DMF (2.4 mg, 5 equiv., V_{DMF}/V_{total} = 10%). The reaction was kept at room temperature for 20 min and analyzed by analytical HPLC (C4 column) using method B (Section 1.2). 3-MPA (5 equiv. compared to Pd-2 complex) was added to quench reaction for additional 10 min at room temperature. Finally modified Myc was purified by RP-HPLC (Method B, Section 1.3) to afford 1.3 mg as white powder (23% yield).

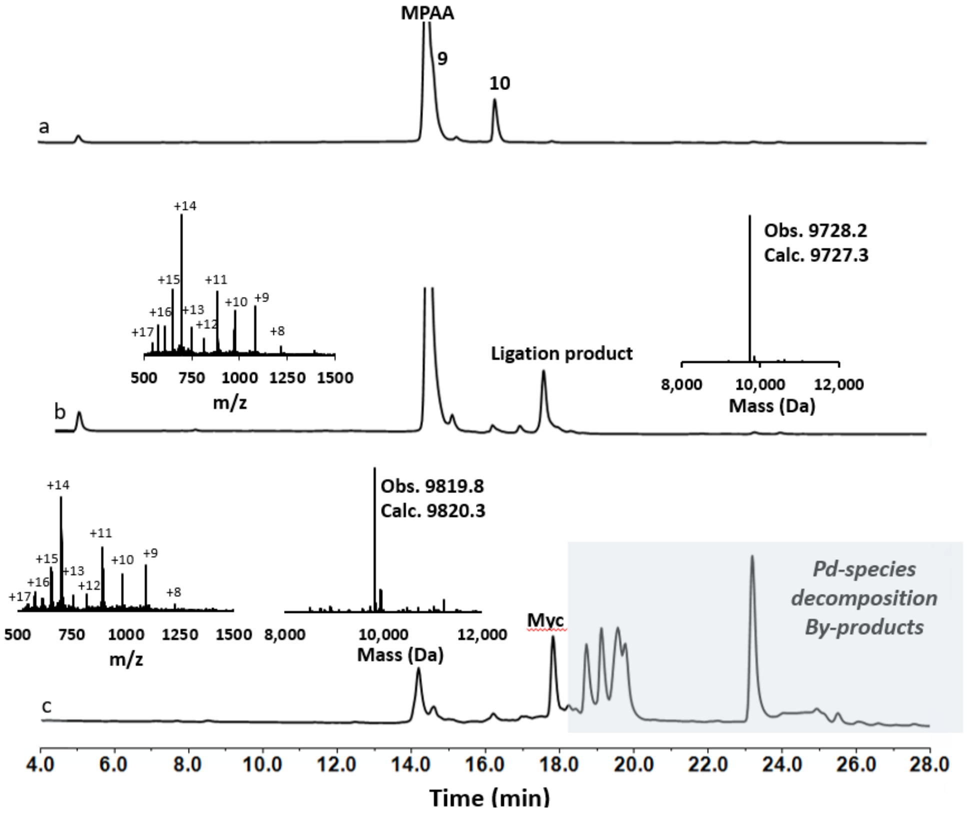

Figure S15. HPLC and MS analysis of the chemical synthesis of Myc. HPLC chromatogram of the UV absorbance at 214 nm. HPLC analysis was carried out with Method B (see section 1.2). Mass-to-charge (m/z) spectrum, and deconvoluted mass spectrum. (a) Ligation at t = 0 for Myc(354-401)-Nbz fragment 9 and Cys-Myc(403-434) fragment 10. (b) Crude ligation reaction at t = 60 min. (c) Crude ligation and S-arylation reaction with Pd-2 at t = 20 min.

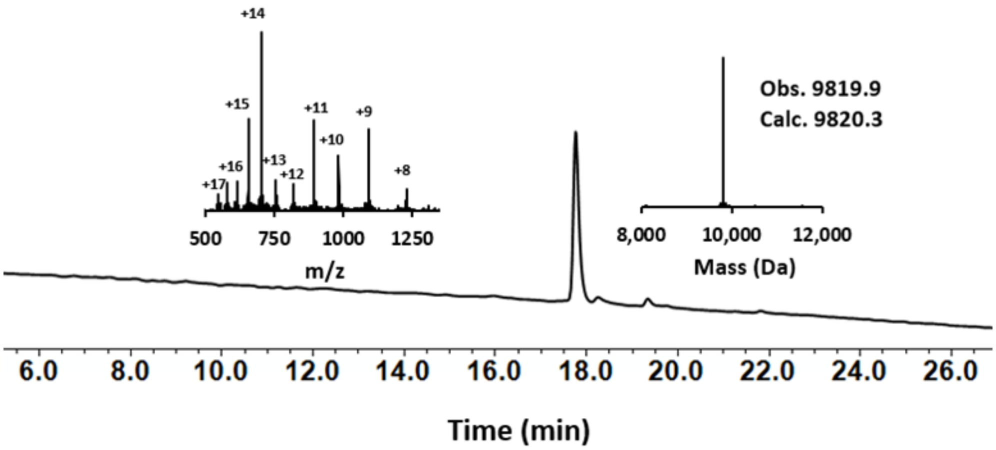

Figure S16. HPLC and MS analysis of Myc. HPLC chromatogram of the UV absorbance at 214 nm. HPLC analysis was carried out with Method B (see section 1.2). Mass-to-charge (m/z) spectrum, and deconvoluted mass spectrum.

6.4 S-arylation of Max followed by byproduct removal via molecular weight filtration

Cys-Max(44-102) 8 (20.0 mg, 2.85 µmol, 1 equiv.) and Max(22-42)-Nbz 7 (11.5 mg, 4.25 µmol, 1.5 equiv.) were dissolved in 6 M Gun·HCl, 200 mM Na_2HPO_4 buffer (1423.0 µL, 2 mM) containing 30 equiv. of MPAA and 15 equiv. of TCEP at pH ~7.2. The reaction mixture was incubated at 37 °C for 60 min. The progress of the reaction was monitored by analytical HPLC by Method B (Section 1.2). After completion of the reaction, ligated Max was purified by RP-HPLC (Method B as described in section 1.3) to afford 12 mg as white powder (44% yield).

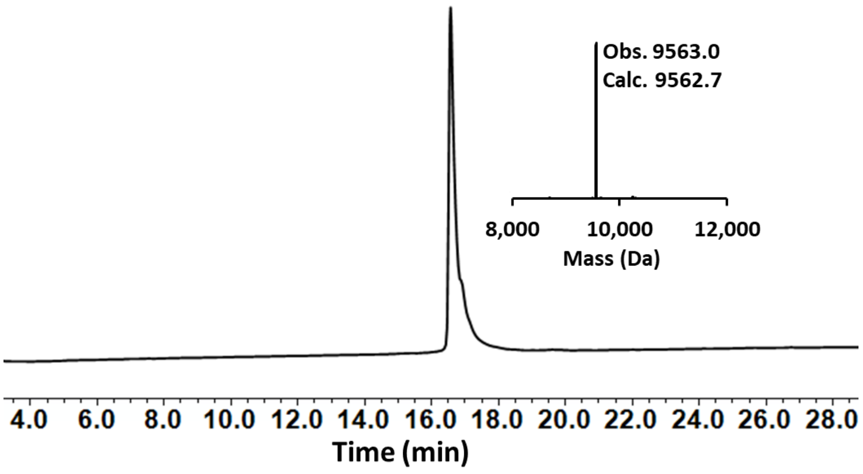

Figure S17. HPLC and MS analysis of ligated Max. HPLC chromatogram of the UV absorbance at 214 nm. HPLC analysis was carried out with Method B (see section 1.2).

To a 1.5 mL Eppendorf tube was added ligated max (90 µL, 0.5 mM, 1.0 equiv.) as a solution in 20 mM Tris (pH 7.2), and Pd-1 (10 µL, 2.5 mM, 5.0 equiv.) as a solution in DMF. The Eppendorf tube was then closed, and the reaction mixture was vortexed and kept at room temperature for 15 min. Next, the Eppendorf tube was opened, and the reaction was treated with 3-MPA (10 equiv. compared to Pd-1) and kept at room temperature for 5 min. The progress of the reaction was monitored by analytical HPLC by Method B (Section 1.2). After completion of the reaction, the crude reaction was desalted by pipetting the reaction mixture into a 3 kDa molecular weight cutoff spin filter (Amicon® Ultra- 0.5 mL, 3K), and was diluted to 0.4 mL using 20 mM Tris buffer (pH 7.2) and concentrated to 0.2 mL at 5000 rpm for 15 min. This process was repeated three times. After that, reaction mixture was collected by reverse centrifuge. The reaction mixture was analyzed by analytical HPLC by Method B (Section 1.2).

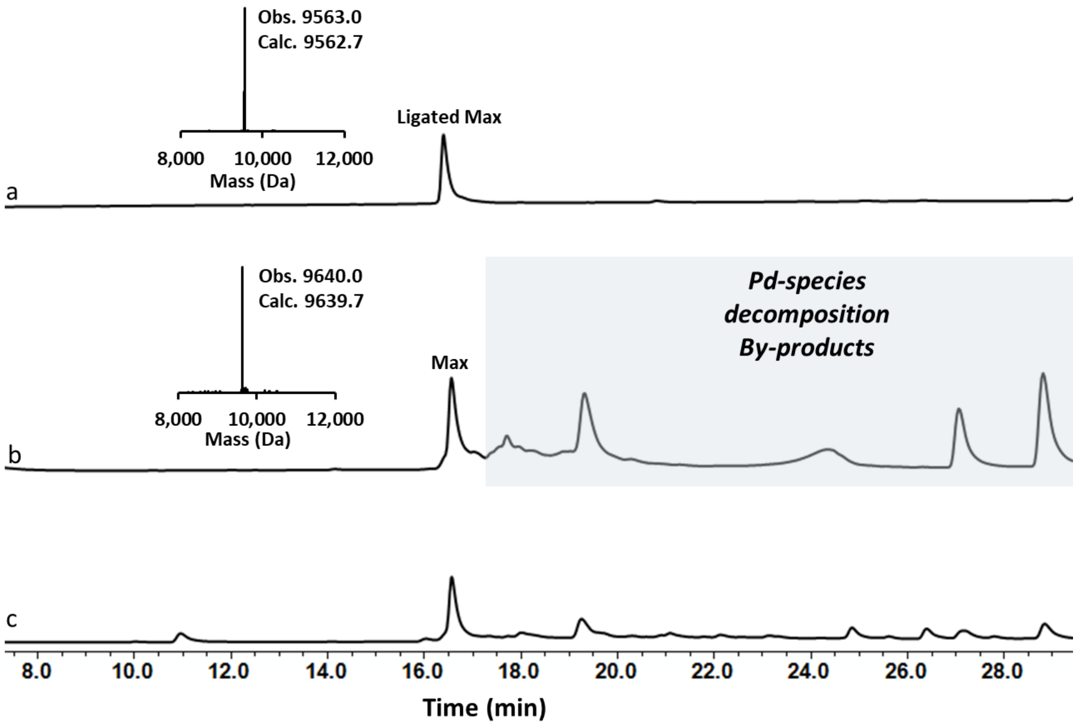

Figure S18. HPLC and MS analysis of modified Max. HPLC chromatogram of the UV absorbance at 214 nm. HPLC analysis was carried out with Method B (see section 1.2). (a) Ligated Max (t = 0). (b) Crude S-arylation reaction with Pd-1 at t = 20 min. (c) Crude S-arylation reaction after molecular weight centrifugal filtration.

7. DNA-Binding Analysis and Electrophoretic Mobility-Shift Assay (EMSA)

To 0.6 mL Eppendorf tube was added the E-box DNA probe (5.0 µL, 10.0 µM) as a solution in 10 mM MES, 150 mM KCl, 1 mM $MgCl_2$, 10% glycerol buffer, 41.0 µL 10 mM MES, 150 mM KCl, 1mM $MgCl_2$, 10% glycerol buffer, and target protein analog (4.0 µL, 50.0 µM; (4 equiv.)) or (8.0 µL, 50.0 µM; (8 equiv.)) as a solution in 10 mM MES, 150 mM KCl, 1 mM $MgCl_2$, 10% glycerol buffer (for homodimers: Max+Max, Myc+Myc). And (2.0 µL, 50.0 µM, Max + 2.0 µL, 50.0 µM, Myc or 4.0 µL, 50.0 µM, Max + 4.0 µL, 50.0 µM, Myc as a solution in 10 mM MES, 150 mM KCl, 1mM $MgCl_2$, 10% glycerol buffer (for heterodimers: Myc+Max). The final reaction concentration of the major reaction components was the following: DNA (1.0 µM): protein (4.0 µM) or protein (8.0 µM). The Eppendorf tube was closed mixed up and down and incubated at room temperature for 1 hour. During the incubation, a 10 % TBE gel, 1.0 mm x 10 well, was pre-run in 1x TBE buffer (1 h, 100V). Next, the DNA-binding activity of each analog was analyzed by EMSA. 5.0 µL of DNA-protein mixture was mixed with 5.0 µL MES buffer and 2.0 µL DNA Loading Dye (6X) and 5.0 µL of each mixture were loaded to the gel, which was run at 100V for 60 min. The gel was washed two times with water for 30 seconds and then stained via SYBR™ Safe DNA Gel Stain in 1xTBE buffer for 30 min at room temperature. Bands were visualized on the GE Healthcare Fujifilm 5100 imager.

8. Octet BioLayer Interferometry Binding Assay (BLI)

Biolayer interferometry (BLI) assays were performed using an Octet R4 System (ForteBio; Menlo Park, CA) in 96 well plates. Streptavidin Octet biosensors (ForteBio; Menlo Park, CA) were dipped into 0.1% BSA, 0.02% Tween-20, 1x PBS (kinetic buffer) for 10 min and then into 200 µL 1 µM of biotinylated E-box DNA probe in the kinetic buffer for the loading step (120 sec). Sensors were then dipped into kinetic buffer for 60 sec. Next, the tips loaded with Max prepared in kinetic buffer at indicated concentrations for 180 sec to obtain the association curve. Finally, the tips were dipped into the kinetic buffer for 240 sec to obtain the dissociation curve. Measurements were carried out at 25 °C. Data were analyzed within the ForteBio Data Analysis software. The association and dissociation curves are fitted with Fortebio Biosystems (global fitting algorism) to obtain the K_D. Kinetic K_D is reported.

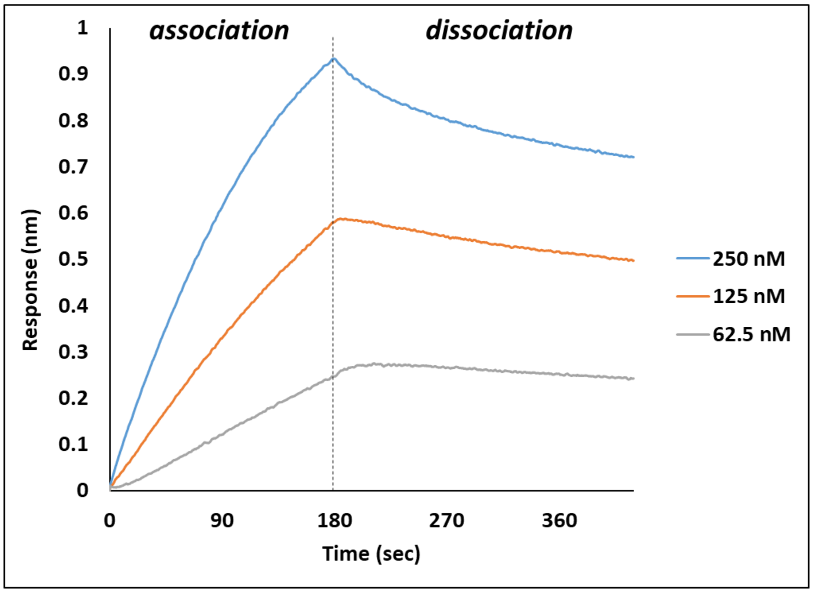

$K_D = 14.8 \pm 0.2$ nM

Figure S19. Biolayer interferometry analysis of synthetic Max binding to E-box DNA probe. Sensorgrams from a biolayer interferometry analysis of Max binding to E-box DNA probe (K_D = 14.8 ± 0.2 nM).

9. NMR data for Pd-1 and Pd-2

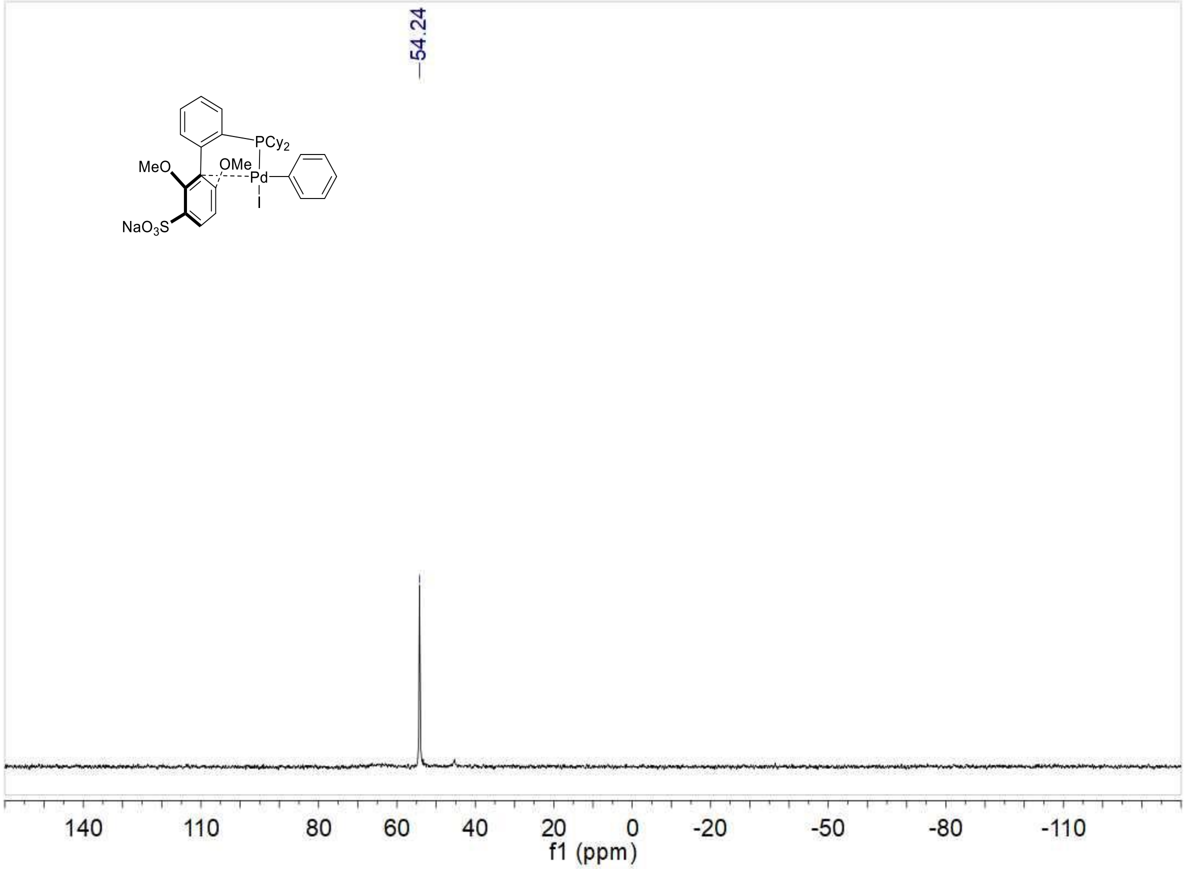

Figure S20. ^{31}P NMR (162 MHz) spectrum of **Pd-1**. NMR of **Pd-1** was taken in *MeOD-d₄*.

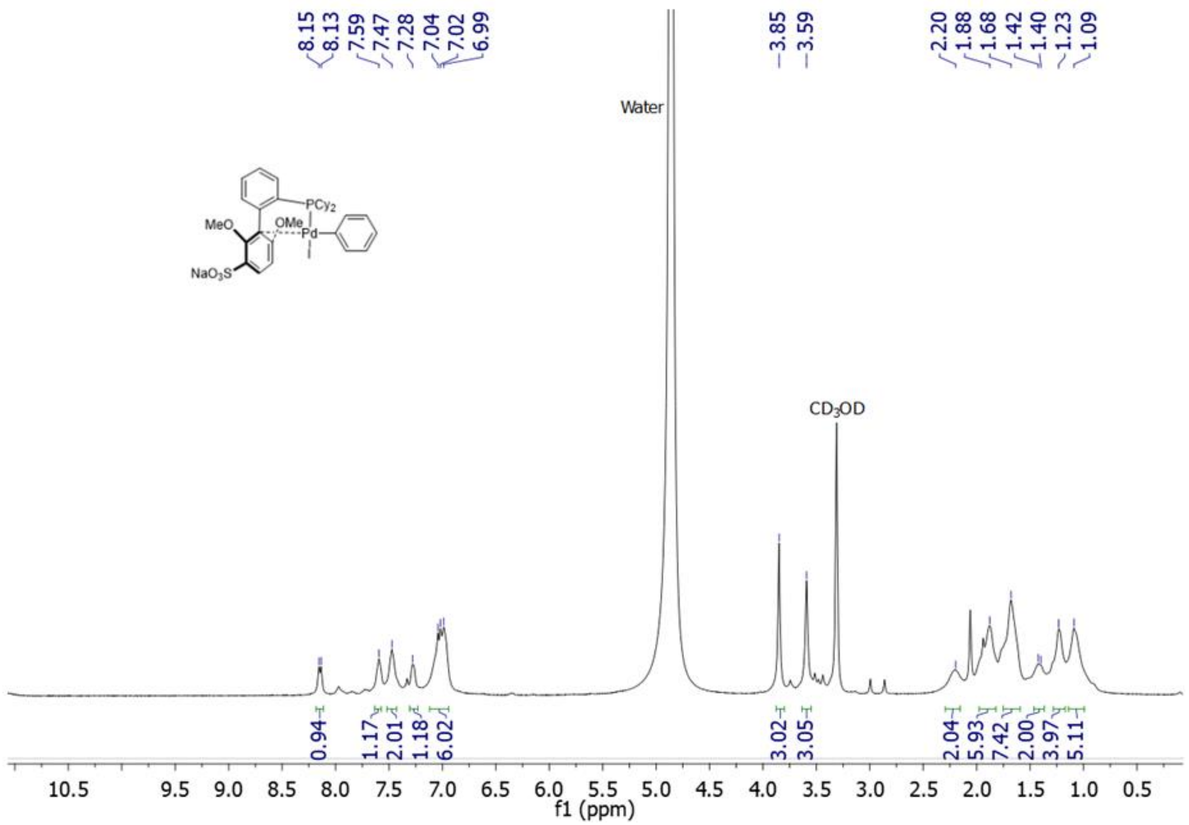

Figure S21. ^{1}H NMR (400 MHz) spectrum of **Pd-1**. NMR of **Pd-1** was taken in *MeOD-d₄*.

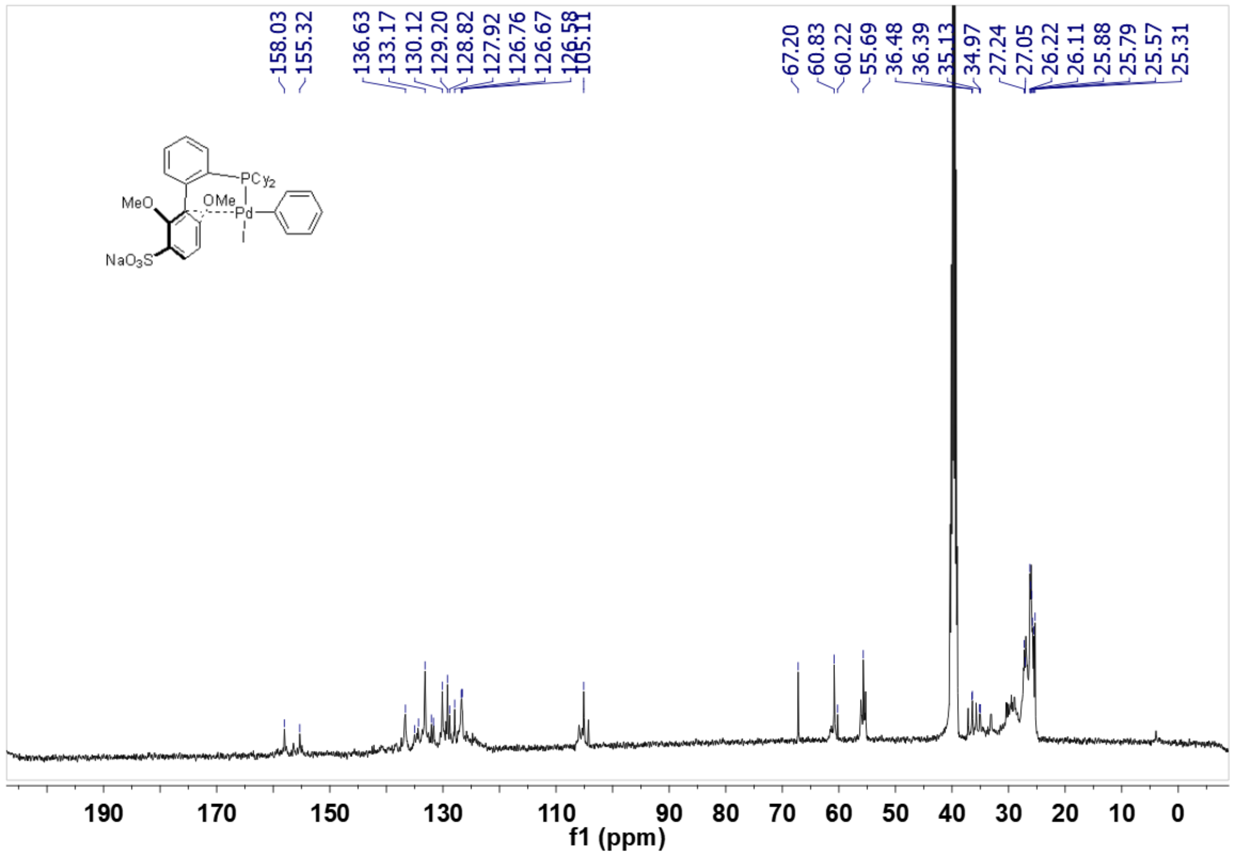

Figure S22. $^{13}C\{1H\}$ NMR (101 MHz) spectrum of **Pd-1**. NMR of **Pd-1** was taken in *DMSO-d₆*.

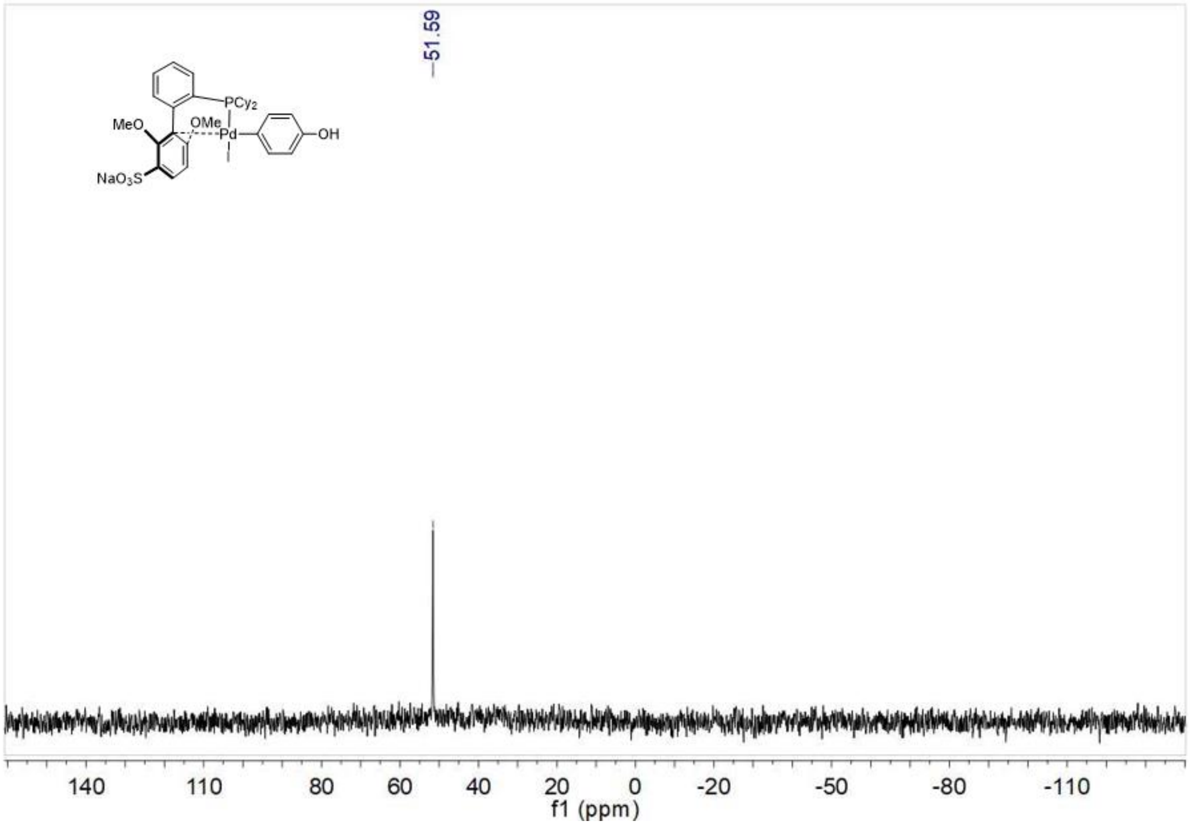

Figure S23. ^{31}P NMR (162 MHz) spectrum of **Pd-2**. NMR of **Pd-2** was taken in *MeOD-d_4*.

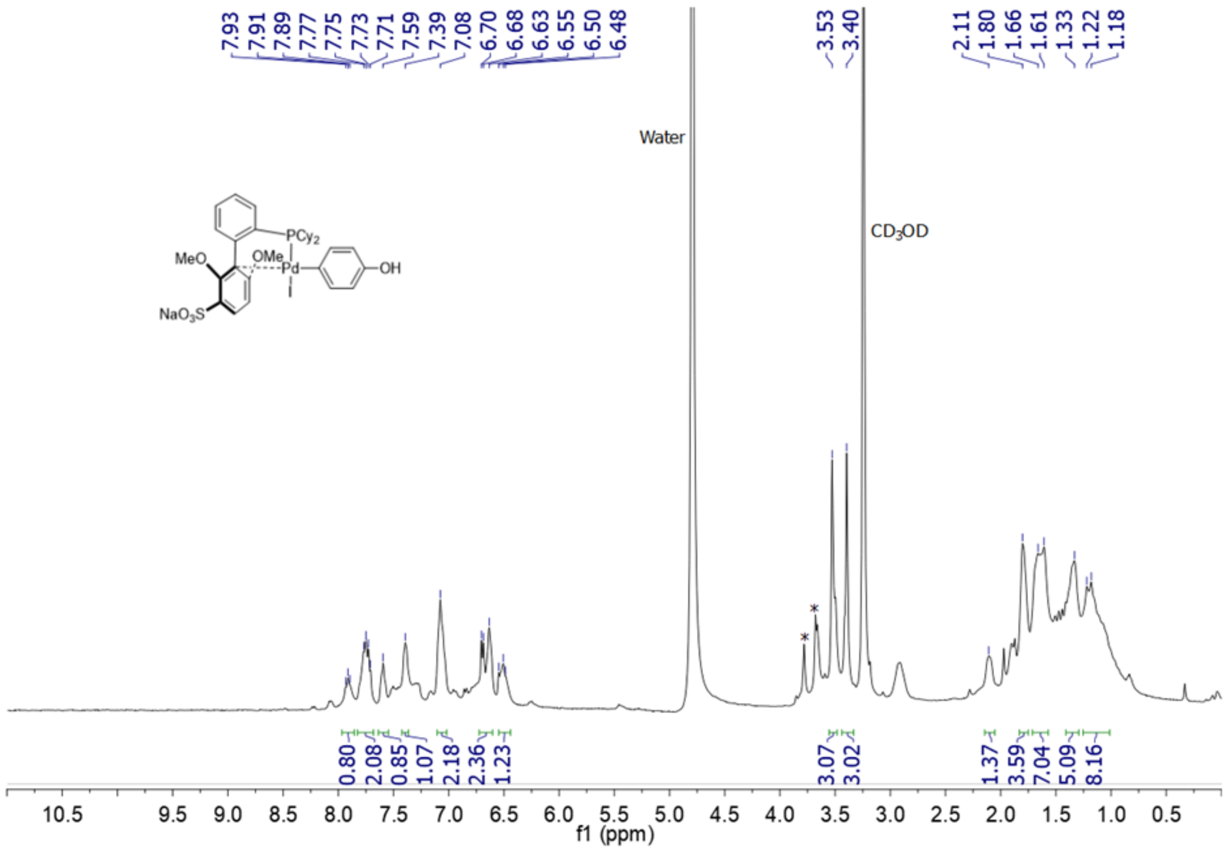

Figure S24. ^{1}H NMR (400 MHz) spectrum of **Pd-2**. NMR of **Pd-2** was taken in *MeOD-d₄*.

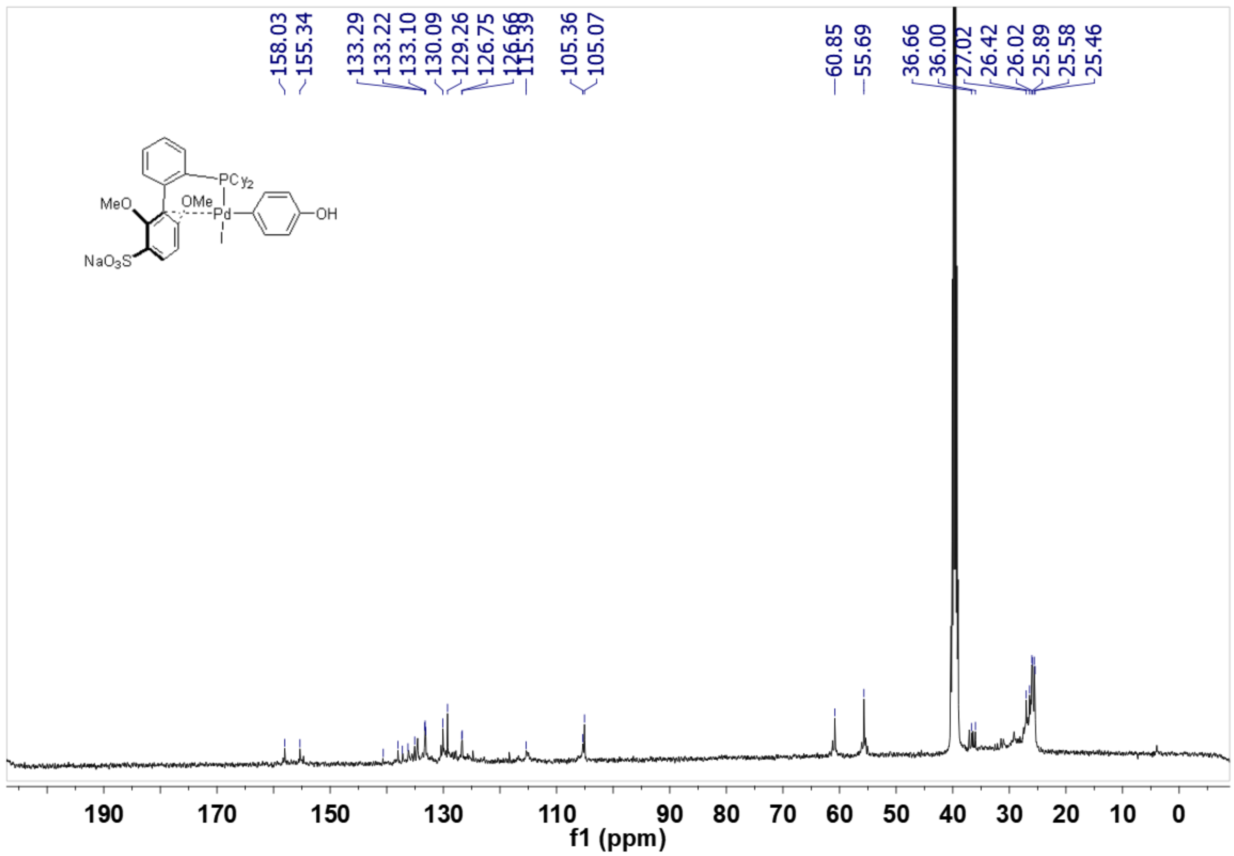

Figure S25. $^{13}C\{1H\}$ NMR (101 MHz) spectrum of **Pd-2**. NMR of **Pd-2** was taken in *DMSO-d_6.*

10. References

1. Dhanjee, H. H.; Saebi, A.; Buslov, I.; Loftis, A. R.; Buchwald, S. L.; Pentelute, B. L. Protein–Protein Cross-Coupling via Palladium–Protein Oxidative Addition Complexes from Cysteine Residues. *J. Am. Chem. Soc.* **2020**, *142*, 9124.

2. Rojas, A. J.; Pentelute, B. L.; Buchwald, S. L. Water-Soluble Palladium Reagents for Cysteine S-Arylation under Ambient Aqueous Conditions. Org. Lett., **2017**, 19 (16), 4263-4266.

3. Vinogradova, E. V.; Zhang, C.; Spokoyny, A. M.; Pentelute, B. L.; Buchwald, S. L. Organometallic Palladium Reagents for Cysteine Bioconjugation. *Nature*, **2015**, *526*, 687.
